# Comparative transcriptomics reveals similarities and differences between astrocytoma grades

**DOI:** 10.1186/s12885-015-1939-9

**Published:** 2015-12-16

**Authors:** Michael Seifert, Martin Garbe, Betty Friedrich, Michel Mittelbronn, Barbara Klink

**Affiliations:** Innovative Methods of Computing, Center for Information Services and High Performance Computing, Dresden University of Technology, Dresden, Germany; Cellular Networks and Systems Biology, University of Cologne, CECAD, Cologne, Germany; Institute of Molecular Systems Biology, Zurich, Switzerland; Institute of Neurology (Edinger Institute), Goethe University, Frankfurt, Germany; Institute for Clinical Genetics, Faculty of Medicine Carl Gustav Carus, Dresden University of Technology, Dresden, Germany; German Cancer Consortium (DKTK), Dresden, Germany; German Cancer Research Center (DKFZ), Heidelberg, Germany

**Keywords:** Astrocytoma grades, Pilocytic astrocytoma, Diffuse astrocytoma, Anaplastic astrocytoma, Glioblastoma

## Abstract

**Background:**

Astrocytomas are the most common primary brain tumors distinguished into four histological grades. Molecular analyses of individual astrocytoma grades have revealed detailed insights into genetic, transcriptomic and epigenetic alterations. This provides an excellent basis to identify similarities and differences between astrocytoma grades.

**Methods:**

We utilized public omics data of all four astrocytoma grades focusing on pilocytic astrocytomas (PA I), diffuse astrocytomas (AS II), anaplastic astrocytomas (AS III) and glioblastomas (GBM IV) to identify similarities and differences using well-established bioinformatics and systems biology approaches. We further validated the expression and localization of Ang2 involved in angiogenesis using immunohistochemistry.

**Results:**

Our analyses show similarities and differences between astrocytoma grades at the level of individual genes, signaling pathways and regulatory networks. We identified many differentially expressed genes that were either exclusively observed in a specific astrocytoma grade or commonly affected in specific subsets of astrocytoma grades in comparison to normal brain. Further, the number of differentially expressed genes generally increased with the astrocytoma grade with one major exception. The cytokine receptor pathway showed nearly the same number of differentially expressed genes in PA I and GBM IV and was further characterized by a significant overlap of commonly altered genes and an exclusive enrichment of overexpressed cancer genes in GBM IV. Additional analyses revealed a strong exclusive overexpression of CX3CL1 (fractalkine) and its receptor CX3CR1 in PA I possibly contributing to the absence of invasive growth. We further found that PA I was significantly associated with the mesenchymal subtype typically observed for very aggressive GBM IV. Expression of endothelial and mesenchymal markers (ANGPT2, CHI3L1) indicated a stronger contribution of the micro-environment to the manifestation of the mesenchymal subtype than the tumor biology itself. We further inferred a transcriptional regulatory network associated with specific expression differences distinguishing PA I from AS II, AS III and GBM IV. Major central transcriptional regulators were involved in brain development, cell cycle control, proliferation, apoptosis, chromatin remodeling or DNA methylation. Many of these regulators showed directly underlying DNA methylation changes in PA I or gene copy number mutations in AS II, AS III and GBM IV.

**Conclusions:**

This computational study characterizes similarities and differences between all four astrocytoma grades confirming known and revealing novel insights into astrocytoma biology. Our findings represent a valuable resource for future computational and experimental studies.

**Electronic supplementary material:**

The online version of this article (doi:10.1186/s12885-015-1939-9) contains supplementary material, which is available to authorized users.

## Background

Astrocytomas are the most common primary brain tumors in the course of life [1]. Molecular origins of astrocytomas are not fully understood. Different studies have identified tumorigenic cells with stem-cell-like properties suggesting that astrocytomas originate from neural stem cells [2, 3]. Astrocytomas are classified by the World Health Organization (WHO) grading system into four histological grades of increasing malignancy [4]. Here, we focus on a comparative analysis of the most frequently occurring astrocytomas (pilocytic astrocytoma, diffuse astrocytoma, anaplastic astrocytoma, glioblastoma) of different degrees of aggressiveness to assess for similarities and differences at the level of individual genes, signaling pathways, molecular subtypes and regulatory networks. This is highly important to better understand the development of specific astrocytomas.

The pilocytic astrocytoma WHO grade I (PA I) is a very slowly growing benign astrocytoma. PA I is the most commonly diagnosed brain tumor in childhood and adolescence [5]. The ten-year overall survival rate of PA I patients is greater than 95 % [1]. The treatment of choice for PA I is gross total resection, but PA I tumors that are inoperable or only partly accessible by surgery represent a therapeutic challenge often showing a serve clinical course [6, 7]. Recent studies have indicated that PA I is predominantly a single-pathway disease driven by mutations affecting the MAPK pathway [5, 7]. In addition, PA I can also display histological features of glioblastoma (GBM IV) including microvascular proliferation and necrosis, but in contrast to GBM IV, these features are not directly associated with increased malignancy of PA I [8]. In rare cases, progression of PA I to more malignant astrocytomas has been observed [9].

In contrast to PA I, astrocytomas of WHO grade II to IV almost exclusively occur in adults. These astrocytomas are characterized by a diffuse infiltrating growth into the surrounding brain tissue that is absent in PA I. Therefore, AS II, AS III and GBM IV are also referred to as diffuse gliomas.

The diffuse astrocytoma WHO grade II (AS II) is a slowly growing invasive semi-benign astrocytoma. AS II is frequently diagnosed in young adults between 20 and 45 years with an average age of 35 years [10]. The diffuse invasive growth of AS II with no clearly identifiable boarder between tumor and normal tissue makes complete surgical resection almost impossible [11]. Recurrences of tumors are observed in most patients after few years with progression to more malignant AS III or GBM IV in many cases [12–14]. The median survival of AS II patients is between five to eight years [15].

The anaplastic astrocytoma WHO grade III (AS III) is an invasively and faster growing malignant astrocytoma. AS III is characterized by increased mitotic activity and more variable size and shape of tumor cells in comparison to AS II [4]. The average age of patients diagnosed with AS III is 45 years. When possible, surgical resection followed by radiotherapy and/or chemotherapy is the treatment of choice. Similar to AS II, progression of AS III to the most malignant GBM IV is frequently observed [13, 14]. The overall five-year survival rate of AS III patients is 24 % [16] and the median survival is between one to four years [17].

The glioblastoma WHO grade IV (GBM IV) is the most malignant astrocytoma [4]. GBM IV is a very fast invasively growing tumor. In contrast to AS III, GBM IV also shows necrosis and/or vascular proliferation. Two genetically distinct GBM IV classes are known: (i) secondary GBMs that develop progressively over several years from less malignant AS II or AS III, and (ii) primary GBMs that develop within few months without prior occurrences of lower grade astrocytomas [12, 13]. Only about 5 % of GBM IV cases are secondary GBMs [18]. Patients diagnosed with a secondary GBM are on average younger than primary GBM patients (45 vs. 62 years) [12]. Primary and secondary GBMs are histologically indistinguishable. IDH mutations in secondary GBMs enable a distinction from primary GBMs at the molecular level [19]. These IDH1 or IDH2 mutations are already present in less malignant AS II and AS III [20]. The treatment of choice is surgical resection in combination with radiation and chemotherapy. This intensive treatment increases the average survival of GBM IV patients to about 15 months [21] compared to 13 weeks for surgery alone [22]. Less than 5 % of patients survive longer than five years [18].

Over the last years, rapid advances in experimental technologies have enabled detailed molecular analyses of large cohorts of different types of astrocytomas that provided new insights into pathological mechanisms [5, 7, 19, 23, 24], molecular subtypes [25–27], alterations of signaling pathways [23, 24, 28], or activities of transcriptional regulatory networks [29–33]. Other studies have focused on the characterization of differences between astrocytoma grades to better understand pathogenic impacts of molecular alterations. Differential expression of immune defense genes in PA I in comparison to AS II with potential indications toward benign behavior of PA I have been reported [34]. Characteristic expression of anti-migratory genes has been found in PA I in comparison to AS II, AS III and GBM IV putatively contributing to the compact, well-circumscribed growth of PA I in contrast to the infiltrative growth of higher-grade astrocytomas [35]. Further molecular markers distinguishing PA I from AS II, AS III and GBM IV have been reported in [36, 37]. A comparative analysis of AS II, AS III and GBM IV has revealed greater regulatory network dysregulation associated with increasing astrocytoma grade [33]. Additionally, mutational patterns associated with the origin and chemotherapy therapy-driven evolution of recurrent secondary gliomas have recently been reported [14]. All these and many other studies have greatly contributed to a better understanding of astrocytoma development hopefully contributing to urgently needed new therapeutic strategies in the near future.

However, most studies have only focused on the identification of differences between astrocytoma grades. This is of course very important to better understand molecular mechanisms associated with aggressiveness of different astrocytoma grades and to reveal novel grade-specific therapeutic targets. On the other hand, still only little is known about commonly altered genes, shared molecular subtypes, common alterations in signaling or metabolic pathways, or activities of major transcriptional regulators. More detailed information about these regulatory mechanisms is also very important to further increase our knowledge about astrocytoma development and may reveal unexpected similarities between astrocytoma grades.

Here, we utilize publicly available molecular data of astrocytomas to systematically characterize similarities and differences of all four astrocytoma grades. In more detail, we characterize transcriptional alterations at the level of individual genes and known molecular pathways. We analyze all four astrocytoma grades for their association with known molecular subtypes and utilize immunohistochemistry to validate Ang2 as a marker gene predicted to distinguish PA I and GBM IV from AS II and AS III. We further determine a regulatory network that distinguishes PA I from AS II, AS III and GBM IV revealing major transcriptional regulators and directly underlying mutations putatively associated with pathobiological differences.

## Methods

No ethical approval was required for this study. All utilized public omics data sets were generated by others who obtained ethical approval.

### Molecular data of PA I

We considered raw gene expression data of 49 PA I and 9 normal cerebellum reference samples (5 fetal and 4 adult samples) available from Gene Expression Omnibus (GSE44971) [38]. We performed stringent quality controls of all expression arrays by reconstructing the hybridization images. We removed three arrays with slight hybridization artifacts. The remaining samples are listed in Additional file 1: Table S1. All corresponding microarrays were normalized using GCRMA [39] with a design file from BrainArray (HGU133Plus2 version 15.0.0). The resulting PA I gene expression data set comprised 47 PA I samples and 8 corresponding normal cerebellum references for which expression levels were measured for 16,973 genes. We further also downloaded processed DNA methylation profiles available for 38 of the considered PA I samples (GSE44684) analyzed in [38]. Tumor-specific DNA methylation profiles were compared to DNA methylation profiles of normal cerebellum samples from four fetal and two adult probes. We refer to [38] for more details. All PA I tumors were diagnosed in children or young adults (Additional file 2: Figure S1) and fulfill all editorial policies (ethical approval and consent, standards of reporting, data availability).

### Molecular data of AS II, AS III and GBM IV

We considered raw gene expression and gene copy number data of AS II, AS III, GBM IV and adult normal brain references from epilepsy patients from the Repository for Molecular Brain Neoplasia Data (Rembrandt, release 1.5.9) [40]. The non-tumor samples from Rembrandt were already used as references for the analysis of AS II, AS III and GBM IV tumors in [41]. We again performed stringent quality controls and removed all patient or reference samples where expression or copy number microarrays had hybridization artifacts. See Additional file 1: Table S1 for considered samples. The remaining gene expression samples were further normalized as previously described for PA I. This resulted in a gene expression data set that comprised 16 AS II, 17 AS III, 45 GBM IV and 21 corresponding normal adult brain references from epilepsy patients for which expression levels were measured for 16,973 genes. Processing of corresponding gene copy number data was more complex (Additional file 2: Text S1). The majority of tumors was diagnosed in older adults. The age at diagnosis tended to increase with the WHO grades of the tumors (Additional file 2: Figure S1). All data sets fulfill the editorial policies (ethical approval and consent, standards of reporting, data availability).

### Identification of differentially expressed genes

We performed t-tests to identify under- and overexpressed genes for each type of astrocytoma (PA I, AS II, AS III, GBM IV) under consideration of the corresponding normal brain references. We corrected for multiple testing by computing FDR-adjusted *p*-values (q-values) for all genes [42] and considered for each type of astrocytoma all genes with q-values below 0.0001 as differentially expressed in tumor compared to normal brain tissue. We further used the sign of the average gene-specific log-ratio of tumor versus normal to specify which of these genes were under- (negative sign) and overexpressed (positive sign) in each specific type of astrocytoma. See Additional file 1: Table S2 for t-test results obtained for all four astrocytoma grades. Further, we note that the considered astrocytoma types represent a heterogeneous group of tumors. PA I is often localized in the cerebellum of children or young adults, whereas AS II, AS III and GBM IV are mainly occurring in the cerebrum of adults. Thus, it is hard to specify a common normal brain reference that would perfectly fit to all astrocytoma types with respect to their different tumor locations and age incidences. Therefore, we decided to analyze all astrocytomas under consideration of the normal brain references that were used in the corresponding initial publications (see [38] for PA I and [40, 41] for AS II, AS III and GBM IV). With the choice of these references we try to control for the heterogeneity of the astrocytoma grades to identify differences in astrocytoma-specific gene expression in comparison to the surrounding normal brain tissue in which these tumors are typically diagnosed. That is, PA I was analyzed with respect to normal cerebellum. Normal brain references from epilepsy patients were considered for the analysis of AS II, AS III and GBM IV. Note that this choice of references does not exclude that some of the differentially expressed genes that distinguish PA I from AS II, AS III and GBM IV may only occur because of expression differences in the corresponding references. However, considering both references, we found a significant positive correlation between average gene expression levels of normal cerebellum and normal brain from epilepsy patients (*r*=0.874, *P*<2.2×10^−16^). This indicates that the majority of genes has very similar expression profiles in both astrocytoma type-specific references. Thus, the used normal brain references should represent a good compromise to account for the location- and age-specific heterogeneity distinguishing PA I from AS II, AS III and GBM IV.

### Molecular subtype classification

We downloaded the Verhaak gene expression signatures of 840 genes (ClaNC840_centroids.xls) available from [25] to determine the similarity of each individual astrocytoma to four known molecular subtypes (neural, proneural, classical, mesenchymal). We identified that 757 of these 840 signature genes were also measured in each of our PA I, AS II, AS III and GBM IV samples. For each of these samples, we first computed for each of the 757 genes its relative expression level (log_2_-ratio) in tumor compared to its average expression in normal brain. Next, we computed the correlations of these 757 sample-specific expression levels with the corresponding expression levels of the four molecular subtypes. We further tested if the correlation of an individual sample with a specific subtype was significantly greater than zero (Pearson’s product moment correlation test). We finally assigned each astrocytoma sample to the Verhaak-subtype with the greatest significant positive correlation (*P*<0.05).

### Molecular signature distinguishing PA I from AS II, AS III and GBM IV

We determined a molecular gene signature that distinguished PA I from AS II, AS III and GBM IV using the previously identified differentially expressed genes. To realize this, we considered each gene that was (i) underexpressed in PA I but not in AS II, AS III or GBM IV, (ii) unchanged in PA I but not in AS II, AS III or GBM IV, or (iii) overexpressed in PA I but not in AS II, AS III or GBM IV. Then, we considered this reversely and determined each gene that was (iv) underexpressed in AS II, AS III or GBM IV but not in PA I, (v) unchanged in AS II, AS III or GBM IV but not in PA I, or (vi) overexpressed in AS II, AS III or GBM IV but not in PA I. All genes that passed one of these criteria showed characteristic expression differences comparing PA I against AS II, AS III or GBM IV. We further only focused on signature genes with strong expression differences and removed all genes with an average gene expression difference below two comparing both classes. This resulted in 1,089 signature genes distinguishing PA I from AS II, AS III and GBM IV. See Additional file 1: Table S3 for obtained signature genes and their average gene expression log-ratios of tumor versus normal.

### Signature-specific regulatory network inference

We considered gene-specific sub-network inference problems to derive a transcriptional regulatory network associated with the expression of molecular signature genes distinguishing PA I from AS II, AS III and GBM IV. Therefore, we focused on the expression levels of *N*=1,089 signature genes in our data set of in total *D*=125 astrocytomas. For each signature gene *i*∈{1,…,*N*}, we assumed that its expression level *e*_*id*_ in an astrocytoma *d*∈{1,…,*D*} can be predicted by a linear combination

(1) $$\begin{array}{*{20}l}  e_{id} &= \sum_{j \in \texttt{TF} \setminus \{i\}} a_{ji} \cdot e_{jd} \end{array} $$

of the expression levels *e*_*jd*_ of transcriptional regulators *j*∈TF∖{*i*} that were part of the molecular signature that distinguishes PA I from AS II, AS III and GBM IV. Here, TF defines the subset of genes in the molecular signature that were annotated as TFs (151 of 1,089). The expression level *e*_*id*_ of each gene *i* in an astrocytoma *d* is given by the log_2_-ratio of the expression level of gene *i* in astrocytoma *d* in comparison to the expression level of gene *i* in the corresponding average normal brain reference. The unknown parameters of this signature gene-specific linear model are given by $\vec {a}_{i} := (a_{\textit {ji}})_{j \in \texttt {TF} \setminus \{i\}}$. Each individual parameter $a_{\textit {ji}} \in \mathbb {R}$ quantifies the impact of the expression level of regulator *j* on the expression level of signature gene *i*: (i) *a*_*ji*_<0 specifies that TF *j* is a putative inhibitor of gene *i*, (ii) *a*_*ji*_>0 defines that TF *j* is a putative activator of gene *i*, and (iii) *a*_*ji*_=0 means that no dependency between *j* and *i* exists. We used lasso (least absolute shrinkage and selection operator) regression [43] in combination with a recently developed significance test for lasso [44] to estimate each *a*_*ji*_ and its corresponding significance for Eq. (1). This enabled us to select the most relevant putative regulators of each signature gene (Additional file 1: Table S4, *P*<5×10^−5^). Details are provided in Additional file 2: Text S2. We further validated the predictive power of the obtained regulatory network on independent astrocytoma data sets (Additional file 2: Text S4, Figure S7) and we also evaluated the putative proportion of included direct TF-target gene interactions (Additional file 2: Text S5, Figure S8). All these validation studies clearly indicated that the regulatory network included relevant TF-target gene links to predict the expression levels of signature genes based on the expression profiles of TFs.

### Gene annotations

We utilized different public resources to create a comprehensive summary of cancer-relevant gene annotations for the analysis of differentially expressed genes. This comprised genes annotated of TFs/cofactors, kinases, phosphatases, signaling pathway genes, metabolic pathway genes, oncogenes, tumor suppressor genes, cancer census genes, and genes essential for cell survival. Details and references are provided in Additional file 1: Table S5. Additional studies of gene functions were done using PubMed (http://www.ncbi.nlm.nih.gov/pubmed) and GeneCards (http://www.genecards.org/).

## Results and discussion

### Transcriptional alterations increase with WHO grade

**Fig. 1:**
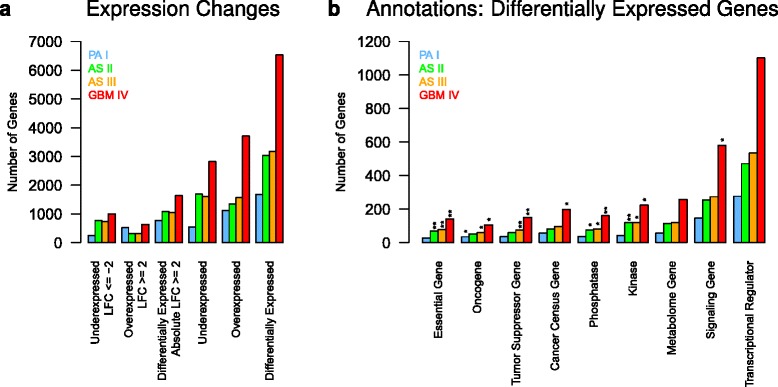
Expression changes and functional categorization of differentially expressed genes for different astrocytoma grades. **a**, Number of differentially expressed genes identified for each type of astrocytoma in comparison to normal brain references at an FDR of 0.0001. An additional log-fold-change cutoff (LFC) of two was used for the first three categories to focus on genes with strong expression changes. **b**, Number of differentially expressed genes annotated in selected functional categories: essential genes, oncogenes, tumor suppressor genes, cancer census genes, phosphatases, kinases, metabolome pathway genes, signaling pathway genes, and transcriptional regulators (see Methods for details). Significant enrichment of genes in a category within a tumor type is represented by ’*’ (*P*<0.05) and ’**’ (*P*<0.01) (Fisher’s exact test)

We first globally analyzed PA I, AS II, AS III and GBM VI and found that the number of differentially expressed genes increased significantly with increasing WHO grade (*r*=0.92, *P*=0.04, Pearson’s product moment correlation). Corresponding statistics are shown in Fig. 1a for each type of astrocytoma. Compared to PA I known to have the best prognosis, AS II and AS III showed a nearly two-fold increase in differentially expressed genes. A nearly four-fold increase was observed for GBM IV representing the most malignant astrocytoma. We also observed that the number of overexpressed genes in PA I was more than two-fold higher than the number of underexpressed genes. This was much more balanced for AS II and AS III. Similar to PA I, GBM IV also showed clearly more over- than underexpressed genes. The global tendencies remained highly similar but the numbers of differentially expressed genes were clearly reduced when we further restricted the identified genes to those with strong expression changes of absolute log_2_-fold-changes greater than two compared to normal brain (Fig. 1a).

Next, we analyzed the identified differentially expressed genes in the context of functional categories or cellular processes known to be involved in cancer. Therefore, we first used data from different public resources to define nine cancer-relevant categories containing genes that are essential for cell survival, oncogenes, tumor suppressor genes, cancer census genes, phosphatases, kinases, metabolome genes, signaling pathway genes, and transcriptional regulators (Additional file 1: Table S5). We then determined for each category the overlap with the differentially expressed genes identified for each type of astrocytoma. Again, we found that the numbers of differentially expressed genes in each category increased significantly with the WHO grades (*r*>0.91, *P*<0.043 for all categories, Pearson’s product moment correlation). A statistic representing the number of differentially expressed genes in each of these categories for each type of astrocytoma is shown in Fig. 1b. Genes essential for cell survival, phosphatases, and kinases were only significantly overrepresented in AS II, AS III and GBM IV. Oncogenes were enriched in PA I, AS III and GBM IV, whereas tumor suppressor genes were only enriched in AS III and GBM IV. Additionally, cancer census genes [45] and genes that were part of known cancer-relevant signaling pathways were only significantly overrepresented in GBM IV. Although not significantly enriched, we observed several differentially expressed metabolic pathway genes, even more differentially expressed cancer-relevant signaling pathway genes, and many differentially expressed transcriptional regulators in all astrocytoma grades with numbers of affected genes again increasing from PA I to GBM IV (Fig. 1b).

Finally, we further extended the previous analysis to distinguish between under- and overexpressed genes (Additional file 2: Figure S2). No enrichment of underexpressed genes was observed for essential and signaling pathway genes in all four astrocytoma grades. Underexpressed genes annotated as oncogenes, tumor suppressor genes, cancer census genes or transcriptional regulators were significantly enriched in PA I. Phosphatases and kinases were significantly overrepresented among underexpressed genes in AS II, AS III and GBM IV. Underexpressed metabolome genes were only significantly enriched in GBM IV. Further, no significant enrichment of overexpressed genes was observed for phosphatases, kinases and metabolome genes in all four astrocytoma grades. Overexpressed oncogenes were significantly overrepresented in AS II and AS III. Transcriptional regulators, tumor suppressors and cancer census genes were significantly enriched for overexpressed genes in AS II, AS III and GBM IV. Overexpressed signaling pathway genes were significantly enriched in all four astrocytoma grades.

### Verhaak classification reveals strong association of PA I with mesenchymal subtype

**Fig. 2:**
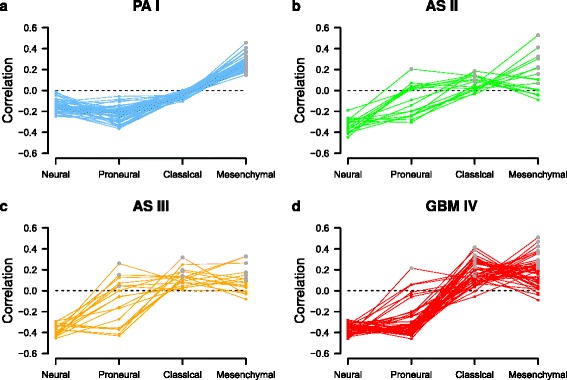
Classification of individual astrocytoma patients according to known molecular subtypes. Classification of PA I, AS II, AS III and GBM IV patients according to molecular subtypes (Neural, Proneural, Classical, Mesenchymal) defined by Verhaak [25]. Correlations between patient-specific expression levels of Verhaak signature genes and each of the four subtype-specific Verhaak signatures were computed for each patient. Colored curves represent the obtained patient-specific correlations. A grey dot within each patient-specific curve highlights the assigned Verhaak-subtype to which the underlying patient had the strongest positive correlation. The subfigures **a** to **d** show the results for individual PA I, AS II, AS III, and GBM IV patients

Classification of astrocytomas according to known molecular subtypes is important to improve treatment decisions and prognosis. Four major subtypes of GBM IV were first revealed in [25] and later also identified in AS II and AS III [27]. This has been widely applied to classify individual AS II, AS III and GBM IV tumors either as neural, proneural, classical or mesenchymal, but so far it has not been tested if one or more of these subtypes are also associated with PA I. Therefore, we used the Verhaak-classifier [25] to compute the correlation between the given signature-specific expression levels of the Verhaak-subtypes and the corresponding gene expression levels of each individual astrocytoma. Correlations of each individual PA I, AS II, AS III and GBM IV tumor with the four Verhaak-subtypes are shown in Fig. 2 and provided in Additional file 1: Table S6.

Interestingly, all PA I tumors showed very homogeneous correlation profiles resulting in a significant association with the mesenchymal subtype (Fig. 2a, *r*>0.14, *P*<2.14×10^−5^ for all PA I, Pearson’s product moment correlation). We further confirmed this observation for an independent PA I cohort [46], where again 40 of 41 PA I tumors were significantly correlated with the mesenchymal subtype (Additional file 2: Figure S3, *r*>0.17, *P*<4.5×10^−7^ for all PA I). The mesenchymal subtype was observed to be strongly associated with cultured astroglial cells that showed high expression of microglia markers [25]. Additionally, PA I was reported to show increased microglia proliferation in comparison to AS II, AS III and GBM IV [47]. This indicates that the strong association of PA I with the mesenchymal subtype may at least in part be explained with the role of the microglia. To analyze this, we first identified that 16 microglia/macrophage marker genes from [48] were part of the Verhaak-classifier (Additional file 1: Table S7). Next, we used these genes and found a significant positive correlation between the average expression levels of microglia/macrophage marker genes in PA I and corresponding mesenchymal subtype expression levels from Verhaak (*r*=0.56, *P*<0.013). This trend was also observed for AS II, AS III and GBM IV average marker expression profiles (*r*>0.58, *P*<0.009) and also for individual AS II, AS III and GBM IV tumors that were not classified as mesenchymal (Additional file 1: Table S7). Thus, additional pathobiological features such as microvascular proliferation and necrosis most likely contribute to the strong association of PA I with mesenchymal subtype.

Microvascular proliferation and necrosis were described as common features of PA I and GBM IV [8]. Also increased necrosis was reported for the mesenchymal subtype [25]. We observed that ANGPT2 (alias ANG2), an endothelial cell marker involved in angiogenesis [49], had significantly higher expression levels in PA I and GBM IV than in AS II or AS III in comparison to normal brain (Additional file 1: Table S2). Interestingly, these astrocytoma grade-specific expression profile of ANGPT2 was highly correlated with that of the endothelial cell marker THBD (*r*=0.86, *P*=0.07), which is part of the Verhaak signature. In contrast to THBD, ANGPT2 is not part of the Verhaak signature, but this positive correlation indicates that microvascular proliferation and necrosis may contribute to the mesenchymal classification obtained for all PA I and many GBM IV tumors. To further test this, we confirmed by immunohistochemistry that PA I and GBM IV showed Ang2-positive endothelial cells (protein expression) in regions with activated blood vessels, a feature that was largely absent in AS II and AS III (Additional file 2: Figure S4, Text S3). We also found that the expression of the mesenchymal marker CHI3L1 [25] was highly correlated with the expression of ANGPT2 (*r*=0.89, *P*<0.06). Thus, this all indicates that several different factors contribute to the strong association of PA I with the mesenchymal subtype. In addition, the micro-environment may have a stronger contribution on these subtype-characteristics than the distinct aggressiveness of mostly benign PA I and highly malignant GBM IV tumor cells.

The Verhaak-classification of AS II, AS III and GBM IV was clearly more heterogeneous revealing few proneural, some classical and many mesenchymal astrocytomas in each class (Fig. 2b–d). The neural subtype was clearly underrepresented in the considered cohorts. Only one PA I tumor from [46] was classified as neural with marginally higher significance than for mesenchymal (Additional file 1: Table S6).

The Verhaak-classification scheme has been further refined by a hypermethylator subtype predominantly observed within a subgroup of proneural astrocytomas [26]. A specific mutation of IDH1 frequently found in AS II, AS III and secondary GBM IV has been shown to be a key driver of this subtype [50]. We used the gene expression signature of the hypermethylator subtype (Table 2 in [26]) to determine the correlation of each of our astrocytoma samples with this subtype. As expected, PA I and the majority of our GBM IV tumors, both typically lacking IDH1 mutations, were negatively correlated with the hypermethylator subtype, whereas the majority of AS II and AS II showed positive correlations (Additional file 2: Figure S5).

### Specific patterns of differential expression characterize similarities and differences of different astrocytomas

**Fig. 3:**
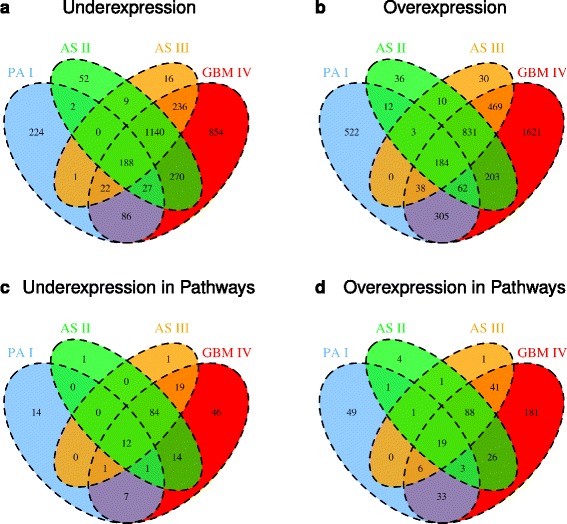
Comparison of genes with altered expression in different astrocytoma grades. Venn diagrams were used to quantify commonalities and differences between differentially expressed genes identified for each type of astrocytoma in comparison to normal brain. **a**, Underexpressed genes. **b**, Overexpressed genes. **c**, Underexpressed cancer signaling pathway genes. **d**, Overexpressed cancer signaling pathway genes

Besides the observed molecular heterogeneity between and within the different astrocytoma types, we next aimed at the identification of core sets of genes that were commonly under- or overexpressed in different astrocytoma subsets. We therefore considered all differentially expressed genes identified for PA I, AS II, AS III and GBM IV and utilized Venn diagrams to quantify the numbers of genes that were exclusively present in specific subsets of these types of astrocytomas (Fig. 3). Expression states of individual genes for all types of astrocytomas are provided in Additional file 1: Table S2. We observed that the number of commonly under- or overexpressed genes in AS II, AS III and GBM IV were substantially increased in comparison to any intersection of PA I with two more malignant astrocytoma grades (Fig. 3a–b, e.g. 1140 under- and 831 overexpressed genes in common between AS II, AS III and GBM IV vs. 27 under- and 62 overexpressed genes in common between PA I, AS II and GBM IV). Additionally, AS II and AS III alone also shared many more commonly under- or overexpressed genes with GBM IV than with PA I (e.g. 270 under- and 203 overexpressed genes in common between AS II and GBM IV vs. 2 under- and 12 overexpressed genes in common between AS II and PA I). Interestingly, there was a strong exclusive overlap of 86 under- and 305 overexpressed genes in common between PA I and GBM IV that contained substantially more genes than observed between PA I and AS II or PA I and AS III. These different general tendencies were also observed when we exclusively focused on known cancer signaling pathway genes (Fig. 3c–d).

We further analyzed which genes were commonly under- or overexpressed in each of the four specific astrocytoma grades and in different subsets of astrocytoma grades (Fig. 3). We also investigated which molecular processes were regulated by subset-specific genes using GOrilla [51]. Since there were so many transcriptomic changes comparing astrocytomas to normal brain tissue, we only report details for some well-known or potentially interesting genes. We further refer to Additional file 1: Table S2 listing the expression states of all genes in specific astrocytoma subsets. In addition, we have summarized all discussed genes that were exclusively differentially expressed in PA I, AS II, AS III or GBM IV in Table 1.

**Table 1:** Selected genes predicted to be differentially expressed in a specific astrocytoma grade

Gene	Chromosome	Band	Expression	Tumor	Annotation
H3F3A	1	q42.12	-	PA I	H3 histone, family 3A
MEIS1	2	p14	-	PA I	Meis homeobox 1
NEUROD1	2	q31.3	-	PA I	neuronal differentiation 1
EOMES	3	p24.1	-	PA I	eomesodermin
ZIC1	3	q24	-	PA I	Zic family member 1
ZIC4	3	q24	-	PA I	Zic family member 4
EGR1	5	q31.2	+	PA I	early growth response 1
EN2	7	q36.3	-	PA I	engrailed homeobox 2
EGR3	8	p21.3	+	PA I	early growth response 3
CDKN2B	9	p21.3	+	PA I	cyclin-dependent kinase inhibitor 2B (p15, inhibits CDK4)
NTRK2	9	q21.33	+	PA I	neurotrophic tyrosine kinase, receptor, type 2
HIF1AN	10	q24.31	+	PA I	hypoxia inducible factor 1, alpha subunit inhibitor
SUV420H1	11	q13.2	-	PA I	suppressor of variegation 4-20 homolog 1 (Drosophila)
KRAS	12	p12.1	-	PA I	Kirsten rat sarcoma viral oncogene homolog
ZIC2	13	q32.3	-	PA I	Zic family member 2
SUZ12	17	q11.2	-	PA I	SUZ12 polycomb repressive complex 2 subunit
SUV420H2	19	q13.42	-	PA I	suppressor of variegation 4-20 homolog 2 (Drosophila)
OLIG1	21	q22.11	+	PA I	oligodendrocyte transcription factor 1
OLIG2	21	q22.11	+	PA I	oligodendrocyte lineage transcription factor 2
ATRX	X	q21.1	-	PA I	alpha thalassemia/mental retardation syndrome X-linked
ZIC3	X	q26.3	-	PA I	Zic family member 3
FAM110C	2	p25.3	-	AS II	family with sequence similarity 110, member C
HEY2	6	q22.31	+	AS II	hes-related family bHLH transcription factor with YRPW motif 2
NR2E1	6	q21	-	AS II	nuclear receptor subfamily 2, group E, member 1
EYA1	8	q13.3	+	AS II	EYA transcriptional coactivator and phosphatase 1
GAS2	11	p14.3	-	AS II	growth arrest-specific 2
DLL3	19	q13.2	+	AS II	delta-like 3 (Drosophila)
CDH4	20	q13.33	-	AS II	cadherin 4, type 1, R-cadherin (retinal)
SHROOM2	X	p22.2	-	AS II	shroom family member 2
AP1AR	4	q25	-	AS III	adaptor-related protein complex 1 associated regulatory protein
CDC27	17	q21.32	-	AS III	cell division cycle 27
PPM1D	17	q23.2	+	AS III	protein phosphatase, Mg2+/Mn2+ dependent, 1D
ZNF24	18	q12.2	+	AS III	zinc finger protein 24
TXN2	22	q12.3	+	AS III	thioredoxin 2
AKT3	1	q44	-	GBM IV	v-akt murine thymoma viral oncogene homolog 3
MDM4	1	q32.1	+	GBM IV	MDM4, p53 regulator
PDGFRB	5	q32	+	GBM IV	platelet-derived growth factor receptor, beta polypeptide
VEGFA	6	p21.1	+	GBM IV	vascular endothelial growth factor A
EGFR	7	p11.2	+	GBM IV	epidermal growth factor receptor
FGFR1	8	p11.23	+	GBM IV	fibroblast growth factor receptor 1
FGFR2	10	q26.13	-	GBM IV	fibroblast growth factor receptor 2
BIRC3	11	q22.2	+	GBM IV	baculoviral IAP repeat containing 3
ERRB2	14	q24.3	+	GBM IV	nuclear receptor
NTRK3	15	q25.3	-	GBM IV	neurotrophic tyrosine kinase, receptor, type 3
BRCA1	17	q21.31	+	GBM IV	breast cancer 1, early onset
AKT2	19	q13.2	+	GBM IV	v-akt murine thymoma viral oncogene homolog 2
SMARCA4	19	p13.2	+	GBM IV	SWI/SNF related, matrix associated, actin dependent regulator of chromatin

Summary of discussed genes that were exclusively observed to be under- or overexpressed in a specific type of astrocytoma. The expression state of a gene in tumor is specified by the ’Expression’ column with ’-’ representing underexpression and ’+’ representing overexpression in comparison to normal brain

**Selected genes exclusively observed in PA I** Considering genes that were exclusively differentially expressed in PA I, we observed several under- (e.g. EN2, EOMES, MEIS1, NEUROD1, ZIC1, ZIC2, ZIC3, ZIC4) and overexpressed (e.g. EGR1, EGR3, OLIG1) TFs involved in brain development. For example, EOMES is involved in neuron division and/or migration [52]. Additionally, three known chromatin remodelers (SUV420H1, SUV420H2, SUZ12) were underexpressed in PA I. In accordance with a recent study [53], ATRX, a biomarker of adult astrocytomas, was underexpressed in PA I. In contrast to AS III and GBM IV, HIF1AN was strongly overexpressed in PA I. Further, CDKN2B, a tumor suppressor for which overexpression has been reported to inhibit cell proliferation and to cause senescence of glioma cells with intact RB pathway [54], was overexpressed. OLIG2, which has been reported to show increased expression in PA I and high-grade gliomas [55], was overexpressed. NRTK2, which has been reported to be highly expressed in low grade (WHO grade I and II) gliomas [56], was overexpressed. Further, KRAS, which plays an important role in cell cycle regulation, was underexpressed. Additionally, H3F3A, which encodes for a histone variant that is predominantly integrated into chromatin of non-dividing cells, was underexpressed.

**Selected genes exclusively observed in AS II** In comparison to PA I and GBM IV, less genes were found to be exclusively differentially expressed in AS II (Fig. 3a–b). FAM110C, which has been reported to be part of a stem cell-related self-renewal signature associated with resistance to chemotherapy [57] and for which overexpression has been shown to promote cell cycle arrest in rats [58], was underexpressed. CDH4, which encodes for a cell-adhesion protein involved in brain segmentation and neural outgrowth, was underexpressed. Underexpression of CDH4 is known to play a role in early tumor progression of colorectal and gastric cancer [59]. NR2E1 (TLX), which is involved in anterior brain differentiation, was underexpressed. Underexpression of NR2E1 has been associated with cancer stem cell death and longer survival of G-CIMP glioma patients [60]. Further, SHROOM2 involved in cell spreading and GAS2 involved in apoptosis were both underexpressed. The transcription factor HEY2 and the Notch ligand DLL3 both known for their functions in neurogenesis and implicated in glioma biology [61] were overexpressed. EYA1, which encodes for a phosphatase and transcriptional coactivator that is involved in DNA repair and which has been associated with glioma tumorigenesis [62], was overexpressed.

**Selected genes exclusively observed in AS III** Like for AS II, only relatively few genes were exclusively differentially expressed in AS III. Interestingly, PPM1D, which is involved in p53-mediated cell cycle arrest, was overexpressed. PPM1D gain-of-function mutations have been reported for brain stem gliomas [63]. Additionally, a PPM1D knock-down has been reported to inhibit proliferation and invasion of glioma cells [64]. Further, AP1AR, which negatively regulates cell spreading, size and motility, was underexpressed. CDC27 (APC3), which is part of the anaphase promoting complex and which is involved in timing of mitosis, was underexpressed. Downregulation of a related component (APC7) of the anaphase promoting complex has been observed in breast cancer with poor prognosis [65]. TXN2, which has been identified to play an important role in the protection of osteosarcomas against oxidant-induced apoptosis [66], was overexpressed. Also ZNF24, which is involved in the maintenance of progenitor cell states in the developing central nervous system, was overexpressed. ZNF24 has further been reported to be involved in the negative regulation of angiogenesis [67].

**Selected genes exclusively observed in GBM IV** Many known cancer genes (e.g. BIRC3, BRCA1, EGFR, ERRB2, PDGFRB, VEGFA) were overexpressed in GBM IV. EGFR signaling has been reported to contribute to radiation and chemotherapy resistance of gliomas [68]. In line with VEGFA overexpression, PDGFRB, which has been reported to enhance glioma angiogenesis in tumor endothelia by promoting pericyte recruitment [69, 70], was overexpressed. Further, MDM4, which has been observed to inhibit a p53-dependent growth control [71, 72], was overexpressed. AKT2, for which underexpression has been reported to induce apoptosis and for which overexpression has been associated with cell survival and invasion of more aggressive gliomas [73, 74], was overexpressed. FGFR1, which has been reported for its increased expression and association with autocrine growth signaling in GBM IV [75], was overexpressed. Further, SMARCA4, which has been observed to have increased expression in gliomas and which is potentially involved in controlling of cell proliferation, migration and invasion [76], was overexpressed. PKG1, which has been reported to promote radioresistance of glioma cells [77, 78], was overexpressed. Further, AKT3, which has recently been reported to inhibit vascular tumor growth [79], was underexpressed. FGFR2, which is frequently found to be underexpressed in primary GBM IV and which has been associated with a poor clinical outcome [80], was underexpressed. NTRK3, which has been reported to show reduced expression in high-grade gliomas due to underlying DNA methylation changes [81], was underexpressed.

**Selected genes in the intersection of PA I, AS II, AS III and GBM IV** Genes commonly under- or overexpressed in PA I, AS II, AS III and GBM VI were involved in cell cycle regulation, differentiation, apoptosis and cell migration. We found that the cyclin-dependent kinase inhibitor CDKN2D was underexpressed and CD44, HIF1A and MAPKAPK3 were overexpressed in all four astrocytoma grades. CD44 is a well-known stem cell marker that has been reported to represent a potential therapeutic target for glioblastoma [82]. HIF1A encodes the alpha subunit of the TF hypoxia-inducible factor-1 (HIF-1), which is one of the master regulators of hypoxia response promoting glioma growth and angiogenesis [83]. MAPKAPK3 is a central integrator of mitogen and stress responses in different MAPK pathways [84]. Interestingly, RB1, a know tumor suppressor controlling the progression through G1 into the S-phase of the cell cycle [85], was overexpressed. Induction of wild-type RB1 has been reported to inhibit tumor growth and tumorigenicity [86]. On the other hand, inactivating mutations affecting the RB pathway have frequently been observed in higher-grade gliomas [85]. This potentially indicates that an overexpression of wild-type RB1 in PA I may contribute to a reduced tumor growth, whereas an exclusive overexpression of CDK4 in concert with RB1 observed for AS II, AS III and GBM IV may counteract the inhibition of tumor growth (see next section for more details to CDK4).

**Selected genes in the intersection of AS II, AS III and GBM IV but not in PA I** Genes commonly under- or overexpressed in AS II, AS III and GBM IV were enriched for cell-cell signaling, cell cycle, differentiation, DNA repair, apoptosis and metabolism. Several known oncogenes (e.g. ABL1, AKT1, MYC, NRAS) and tumor suppressor genes (e.g. ATM, BCL10, TP53) were overexpressed in all three astrocytoma types. AKT1 has been found to enhance proliferation and invasion of glioma cells [87]. Overexpression of NRAS that increased with glioma grade was observed in [88]. Overexpression and different cellular locations of TP53 have been reported for primary and secondary glioblastomas impacting on vasculature control and tumorigenesis [89]. Overexpression of TP53 has also been associated with shorter progression free survival in malignant gliomas [90]. Further, also CDK4 and RAF1 were overexpressed. CDK4 overexpression has been reported to induce hyperploidy and to counteract senescence of cultured mouse astrocytes [91]. Astrocyte-specific overexpression of CDK4 in transgenic mouse lines has been observed to provide cell growth advantages in concert with TP53 pathway alterations [92]. Consecutive RAF1 activation has been reported to induce glioma formation in mice [93]. Moreover, also IDH1 was overexpressed. Interestingly, the overexpression of IDH1 in gliomas has recently been reported to have different impacts on chemotherapy response. Wild-type IDH1 was associated with resistance, whereas mutant-IDH1 showed enhanced sensitivity to therapy [94]. MAP2K4, which has been reported to inhibit tumor cell invasion in lung cancer [95], was strongly underexpressed. Further, also MAP2K1, which is involved in the regulation of many cellular processes including proliferation, differentiation and apoptosis, and also MKRN1, which has been observed to stimulate apoptosis under stress conditions [96], were both underexpressed.

**Selected genes in the intersection of AS III and GBM IV but not in PA I and AS II** Genes commonly under- or overexpressed in AS III and GBM IV were involved in cell migration, cell cycle, DNA repair, chromatin organization, angiogenesis and metabolism. HIF1AN (FIH-1), an inhibitor of the previously reported HIF-1, was underexpressed. HIF1AN is involved in hypervascularization and survival of glioma cells under hypoxic conditions and may represent a potential therapeutic target [97]. EZH2, a member of the polycomb-group family involved in the control of DNA methylation [98] and histone H3K27 trimethylation [99] over cell generations, was overexpressed. Also VEGFB involved in blood vessel survival [100] and CDC20 contributing to survival of glioma initiating cells [101] were overexpressed. Further, SOX2, a marker for undifferentiated and proliferating cells observed to show expression levels that increase with the glioma grade [102] and reported to regulate genes and pathways associated with malignancy of stem-like and differentiated glioma cells [103], was overexpressed. TACC3, a potential oncogene overexpressed in a grade-specific manner [104] and observed as fusion partner of FGFR3 in glioblastomas [105], was overexpressed. Moreover, IDH2 was overexpressed. Interestingly, another study has associated the overexpression of a point-mutated IDH2 (IDH2R172K) with increased radio sensitivity, reactive oxygen metabolism, suppression of tumor growth and migration in glioma cell lines compared to wild-type IDH2 [106]. Thus, the underlying mutational status of IDH2 may influence tumor aggressiveness of AS III and GBM IV.

### Transcriptional alterations of individual signaling pathways typically increase with WHO grade

**Fig. 4:**
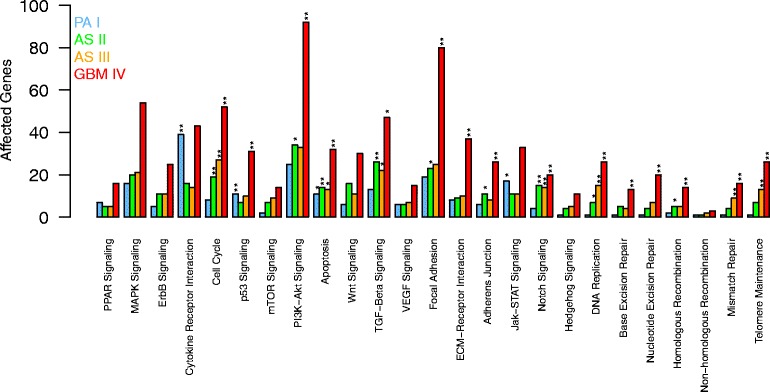
Characteristic patterns of overexpression in signaling pathways distinguishing different astrocytoma grades. Number of overexpressed genes in each known cancer-relevant signaling pathway are shown for each type of astrocytoma (PA I, AS II, AS III, GBM IV). Significant enrichment of overexpressed genes in a pathway within a tumor type is highlighted by ’*’ (*P*<0.05) and ’**’ (*P*<0.01) (Fisher’s exact test)

Next, we focused on individual cancer-relevant signaling pathways and determined corresponding differentially expressed genes for each type of astrocytoma. Figure 4 shows the numbers of overexpressed genes in known cancer signaling pathways representing major differences and some similarities between individual astrocytoma types. We observed strong differences in the number of overexpressed genes for nearly all pathways with gradual increases from PA I to GBM IV. This trend was also observed for the majority of signaling pathways considering underexpressed genes, except for the DNA replication pathway and all DNA repair pathways that both only showed very few or no underexpressed genes in all four astrocytoma grades (Additional file 2: Figure S6). Focusing on overexpression (Fig. 4), especially genes involved in cell cycle, PI3K-AKT, TGF-Beta, focal adhesion, notch, DNA replication and DNA repair pathways were significantly affected by overexpression in AS II, AS III or GBM IV. Genes involved in the regulation of apoptosis were enriched in all four astrocytoma types.

Interestingly, the cytokine-cytokine receptor interaction pathway did not not follow the general trend that the numbers of overexpressed genes systematically increased from PA I to GBM IV. This pathway showed nearly the same proportion of overexpressed genes in PA I as in GBM IV, whereas the proportions of overexpressed genes in AS II and AS III were consistently only approximately half as large as for PA I and GBM IV (Fig. 4). This atypical behavior also strongly contributed to significant exclusive overlaps between PA I and GBM IV comparing under- and overexpressed genes (purple subsets in Fig. 3c–d: 7 underexpressed genes with *P*<6.2×10^−6^ and 33 overexpressed genes with *P*<1.7×10^−8^, Fisher’s exact test). We additionally note that the p53 pathway and the Jak-STAT pathway showed both a very similar behavior comparable to those of the cytokine-cytokine receptor pathway (Fig. 4).

### Highly overlapping expression patterns of cytokine-cytokine receptor interaction pathway between PA I and GBM IV, but only GBM IV is enriched for known cancer genes

**Fig. 5:**
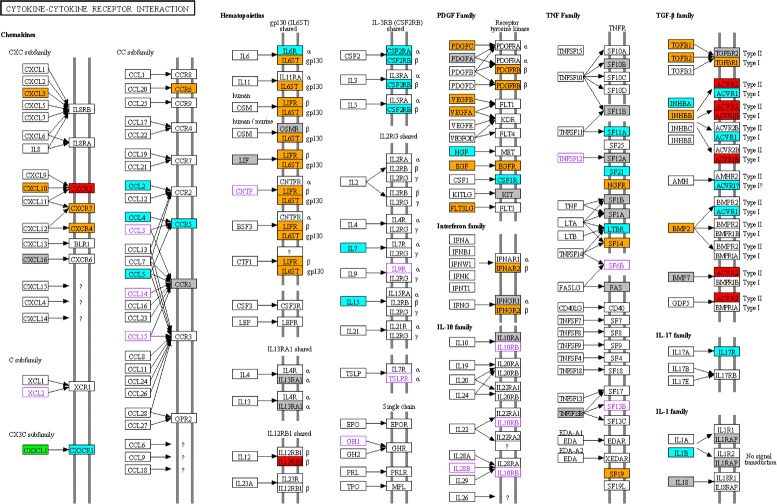
Transcriptional alterations of cytokine-cytokine receptor interaction pathway genes comparing PA I and GBM IV. Representation of under- and overexpressed cytokine-cytokine receptor interaction pathway genes identified in PA I and GBM IV in comparison to normal brain tissue. The basic pathway map (hsa:04060) was generated using KEGG [130] and manually modified. Genes are colored according to their observed expression level (i) genes only overexpressed in PA I (*light blue*), (ii) genes only underexpressed in GBM IV (*red*), (iii) genes only overexpressed in GBM IV (*orange*), (iv) genes commonly overexpressed in PA I and GBM IV (*grey*), except for KIT that was underexpressed in both tumor types, (v) CX3CL1 overexpressed in PA I and underexpressed in GBM IV (*green*), (vi) genes that were not present on the microarray (*purple*), and (vii) genes with unchanged expression levels (*white*)

We observed similar proportions of overexpressed genes in the cytokine-cytokine receptor interaction pathway for PA I and GBM IV (Fig. 4). Cytokines are intracellular signaling proteins that are important regulators of immune response, cell growth, differentiation, metastasis, apoptosis and angiogenesis [107–109]. Some alterations of expression levels of specific cytokines, their corresponding receptors and links to their potential role in brain tumor development have already been reported for benign and malignant astrocytomas more than a decade ago [110–112]. In addition, different chemokines and chemokine receptors were found to contribute to glioma cell survival, migration and invasion [113–118]. We therefore focused on individual genes in the cytokine-cytokine receptor interaction pathway to provide a comprehensive overview of differentially expressed genes comparing PA I and GBM IV. A representation of the cytokine-cytokine receptor interaction pathway highlighting exclusively affected and commonly altered genes is shown in Fig. 5. We found a significant overlap of commonly observed under- and overexpressed genes in the cytokine-cytokine receptor interaction pathway comparing PA I and GBM IV (overlap: 20 genes, 1 underexpressed, 19 overexpressed genes, *P*<2.5×10^−42^, Fisher’s exact test). We further identified genes that were only differentially expressed in PA I (1 under- and 20 overexpressed genes) or in GBM IV (5 under- and 24 overexpressed genes) alone. Only genes that were exclusively overexpressed in GBM IV were significantly enriched for known cancer genes [45] (*P*<4.2×10^−5^, Fisher’s exact test). These genes were mainly assigned to the CXC chemokine, hematopoietin, PDGF or TGF-Beta pathway subfamilies of the cytokine-cytokine receptor interaction pathway (Fig. 5). This included genes such as EGFR, PDGFRB, TNFRSF14 or VEGFA previously associated with aggressiveness, invasion and poor outcome of GBM IV [25, 119, 120].

### Differences in CX3CL1 expression between PA I and AS II, AS III and GBM IV may contribute to absence or presence of glioma cell invasion

Interestingly, CX3CL1 (also known as fractalkine or neurotactin), a member of the cytokine-cytokine receptor interaction pathway (Fig. 5) encoding for a chemokine, showed a characteristic expression pattern distinguishing PA I from AS II, AS III and GBM IV (Additional file 1: Table S2). The soluble form of the CX3CL1 protein is a potent chemoattractant of T-cells and monocytes, while the cell-surface-bound form promotes strong adhesion of those leukocytes [121]. CX3CL1 implements its adhesive and migratory functions by interacting with the chemokine receptor CX3CR1 [122]. The roles of CX3CL1 and CX3CR1 in glioma invasion and progression have been reviewed for malignant astrocytomas in [117]. Potential contributions of both genes to suppress an invasive phenotype in PA I have not been studied so far.

We found that CX3CL1 and CX3CR1 were overexpressed in PA I in comparison to normal brain tissue, whereas we further observed strong underexpression of CX3CL1 and unchanged expression of CX3CR1 in AS II, AS III and GBM IV (Additional file 1: Table S2). In accordance, CX3CR1 has been reported to be expressed in gliomas [123], and CX3CL1 has been reported to reduce neuronal migration by increasing cell adhesion [124]. Potentially, a similar CX3CL1-induced mechanism in PA I may contribute to the absence of infiltrative growth typically observed for AS II, AS III and GBM IV [125]. This hypothesis is supported by the finding that the inhibition of CX3CL1 strongly increased glioma cell invasion suggesting that functionally active CX3CL1 counteracts an invasive phenotype [116]. Additionally, they also reported that TGFB1 negatively influenced the expression of CX3CL1 facilitating glioma cell detachment and dispersion. In agreement with our hypothesis, the expression levels of CX3CL1 were negatively correlated with those of TGFB1 (*r*=−0.98, *P*<0.01). We observed overexpression of TGFB1 in AS II, AS III and GBM IV, whereas TGFB1 expression was unchanged in PA I in comparison to normal brain tissue (Additional file 1: Table S2).

### A transcriptional signature distinguishes PA I from AS II, AS III and GBM IV

**Fig. 6:**
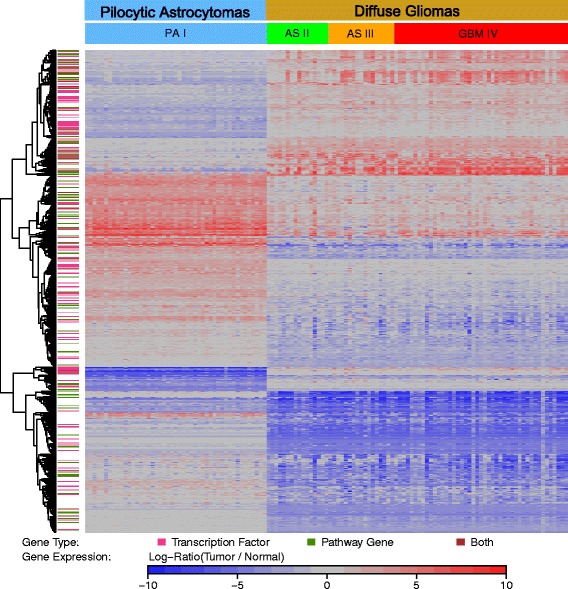
Molecular signature distinguishing PA I from AS II, AS III and GBM IV. The heatmap shows the expression levels of 1,089 genes (*rows*) with strong expression differences between PA I and AS II, AS III and GBM IV for individual tumor patients (*columns*). Expression levels are displayed as log-ratios comparing gene expression levels in tumor to normal brain. Underexpressed genes are displayed in blue, unchanged expressed genes in grey, and overexpressed genes are displayed in red. Genes were clustered according to their similarity of expression levels across all tumor samples. The color code at the left side highlights genes that are TFs (*purple*), signaling pathway genes (*green*), or both (*brown*)

Besides some similarities, our previous studies clearly indicated the existence of systematic differences between PA I and AS II, AS III and GBM IV supporting the finding that both classes represent different pathobiological entities [126]. To further investigate this, we determined a molecular signature comprising 1,089 differentially expressed genes distinguishing PA I from AS II, AS III and GBM IV (Fig. 6, Additional file 1: Table S3). This signature included all under- and overexpressed genes from PA I that did not show the same expression state in AS II, AS III or GBM IV. Vice versa, this signature also included each gene that was identified as under- or overexpressed in AS II, AS III or GBM IV but which did not show the same expression state in PA I. Clusters of genes that were under- or overexpressed in one class but not in the other are clearly visible characterizing differences between PA I and AS II, AS III and GBM IV (Fig. 6). A gene annotation analysis (Additional file 1: Table S3) further revealed that nearly 14 % of the signature genes were annotated as TFs (151 of 1,089), about 10 % were part of known cancer-relevant signaling pathways (111 of 1,089), about 5 % were known cancer genes (55 of 1,089) and about 3 % were part of metabolic pathways (34 of 1,089).

### A regulatory network is associated with expression differences between PA I and AS II, AS III and GBM IV

**Fig. 7:**
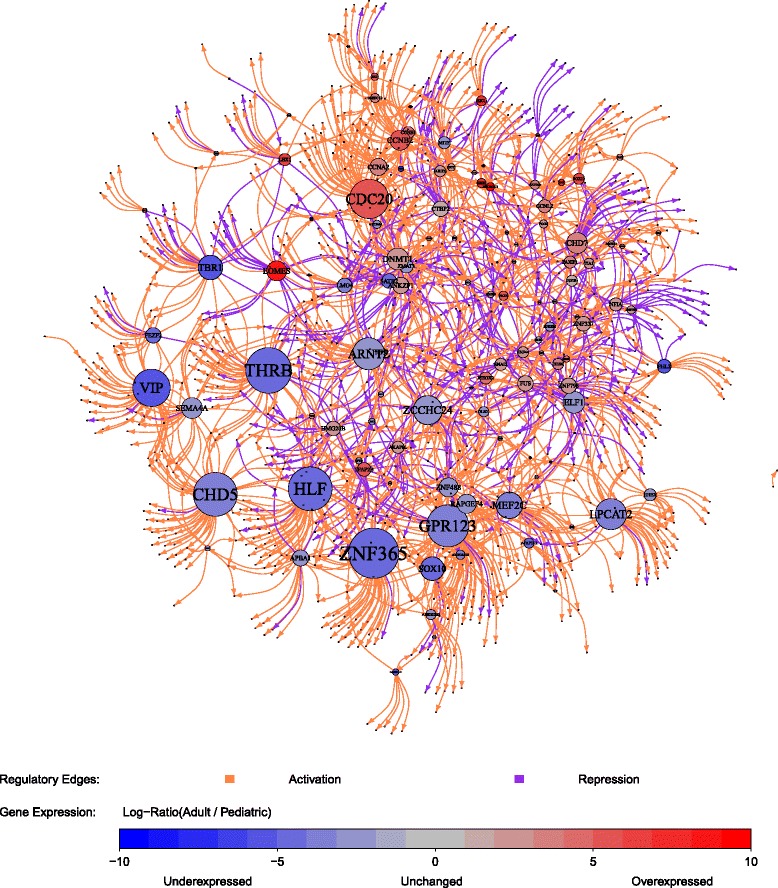
Transcriptional regulatory network distinguishing PA I from AS II, AS III and GBM IV. TFs are displayed by labeled circles. The circle size increases with the number of outgoing regulatory edges to other signature genes highlighting major regulators by large circles. The color coding of the circle represents the average expression level of the corresponding gene in AS II, AS III and GBM IV diagnosed in adults relative to PA I diagnosed in children and young adults: underexpressed (*blue*) and overexpressed (*red*) in adult astrocytomas. Inferred regulatory dependencies between TFs and signature genes are displayed by directed edges: activator (*orange*) and repressor (*purple)*

Next, we used the 151 differentially expressed TFs from the molecular signature (Fig. 6, Additional file 1: Table S3) to learn a transcriptional regulatory network that best explained expression changes of all signature genes distinguishing PA I from AS II, AS III and GBM IV (Fig. 7, Additional file 1: Table S4). This network contained for each individual signature gene those TFs that may act as putative regulators of this gene. The regulatory network was extremely sparse containing only 1,558 out of 164,439 theoretically possible regulatory links from TFs to signature genes. We observed more than three times more activator than repressor links in the network (1,195 vs. 363). Nine TFs did not have any outgoing regulatory links to other signature genes, and no putative regulators were identified for 83 signature genes.

Still, as expected, the obtained regulatory network was highly predictive for the expression levels of signature genes in our astrocytoma data set used to learn the network (Additional file 2: Figure S7a). We further used the obtained regulatory network to predict expression changes of signature genes in three independent brain tumor cohorts (41 PA I from [46], 465 low grade gliomas including 50 AS II and 104 AS III from TCGA LGG, 553 GBM IV from TCGA GBM [23], see Additional file 2: Text S4 for details). We observed that the regulatory network was very predictive for the vast majority of signature genes (Additional file 2: Figures S7b–d). We also analyzed the proportion of putative direct TF-target gene interactions by comparing predicted target genes of TFs in the regulatory network to target genes predicted by TF-based motif search in promoter sequences of signature genes (see Additional file 2: Text S5 for details). We observed significant overlaps of network- and motif-based target genes for many TFs, but there were also TFs with only little or no overlaps (Additional file 2: Figure S8). All these tests indicated that the regulatory network contained relevant TF-target gene links to enable the prediction of signature gene expression levels.

### Expression changes of hub regulators characterize differences between PA I and AS II, AS III and GBM IV

We next utilized the obtained signature-specific regulatory network to identify central hub TFs with many outgoing links to other signature genes. These hub regulators are represented by large nodes in Fig. 7. The majority of these TFs had on average lower expression levels in AS II, AS III and GBM IV than in PA I (blue nodes). A smaller proportion of hub TFs had higher expression levels in AS II, AS III and GBM IV than in PA I (red nodes). Many of these hub TFs were part of three major functional categories: (i) TFs involved in apoptosis, cell proliferation, cell cycle and DNA repair (CCNA2, CCNB1, CCNB2, CDC20, CHD5, GPR123, MEF2C, NEUROD1, VIP, ZNF365), (ii) TFs involved in chromatin remodeling, histone modifications and DNA methylation (CHD5, DNMT1, EZH2, JARID2), and (iii) TFs involved in brain development and differentiation (ARNT2, CHD5, DNMT1, ELF1, EOMES, HLF, JARID2, LHX1, MEF2C, NEUROD1, OLIG1, SOX10, SOX11, THRB, TBR1, VIP, ZIC1, ZIC3).

Next, we studied the hierarchy of TFs in the regulatory network to identify signature-specific hub TFs that had many regulatory links to other TFs. We found that several TFs had clearly increased numbers of outgoing links to other TFs (Additional file 2: Figure S9). Six TFs had more than five outgoing regulatory links to other TFs (CCNL2, GPR123, ZCCHC24, TBR1, ZNF300, ZNF337). CCNL2 encodes for a cyclin involved in the regulation of splicing, apoptosis and cell growth [127]. GPR123 is a member of the adhesion family of G-protein coupled receptors mutated in leukemia [128]. TBR1 encodes for a T-box TF required for normal brain development expressed in post-mitotic cells [129]. Nothing was known in the literature about the functions of ZCCHC24, ZNF300 and ZNF337 so far. We analyzed their network-target genes to learn more about their putative functions. This suggested that ZCCHC24 is involved in the regulation of the cell cycle and of cell-cell interactions. ZNF300 might act on developmental processes impacting on DNA and histone methylation patterns. ZNF337 might contribute to genomic and epigenomic integrity.

### Mutations affecting TFs contribute to differences between PA I and AS II, AS III and GBM IV

**Fig. 8:**
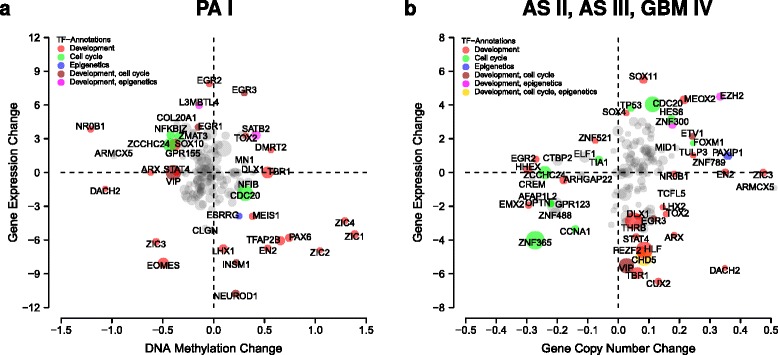
Epigenomic and genomic mutations associated with TF expression changes distinguishing PA I from AS II, AS III and GBM IV. TFs are displayed by circles. The circle size increases with the number of outgoing regulatory edges to other signature genes highlighting major regulators by large circles. Names of TFs strongly deviating from the center are shown in black. TFs belonging to selected annotation categories are highlighted by colored circles. Gene-specific changes in expression, DNA methylation or copy number are quantified by log-ratios comparing tumor to normal brain tissue. A log-ratio close to zero indicates no change in tumor, whereas a strong deviation from zero indicates a change. **a**, Average DNA methylation changes associated with TFs plotted against their average expression profiles in PA I. **b**, Average copy changes of TFs plotted against their average expression profiles in AS II, AS III and GBM IV

To further characterize how genomic and epigenomic mutations may have contributed to expression differences of TFs between PA I and AS II, AS III and GBM IV, we analyzed the individual signature-specific TFs for alterations of DNA methylation levels or gene copy number mutations in comparison to normal tissue. Gene copy number mutations are typically absent in PA I, but changes of DNA methylation patterns within gene bodies or up- and downstream of transcription start sites have been reported [38]. In contrast to PA I, deletions and amplifications of individual genes are typically present in AS II, AS III and GBM IV [40]. DNA methylation profiles were available for the majority of our PA I tumors (38 of 47) and gene copy number profiles were available for all our AS II, AS III and GBM IV tumors. We therefore analyzed the expression of individual signature-specific TFs in relation to directly underlying mutations (Fig. 8).

We found for PA I that TFs with altered expression and/or altered DNA methylation levels were part of three major functional categories (Fig. 8a): (i) TFs involved in development and differentiation (e.g. EN2, EOMES, DMRT2, NR0B1), (ii) TFs involved in cell cycle control, proliferation and apoptosis (e.g. CDC20, NFKBIZ, ZCCHC24), and (iii) TFs involved in chromatin remodeling and DNA methylation (ESRRG, L3MBTL4, SATB2). Several TFs were strongly under- or overexpressed in PA I without strong directly underlying DNA methylation changes (e.g. EGR2, INSM1, LHX1, NEUROD1). None of the central hub TFs in Fig. 7 showed strong expression changes in PA I in response to directly underlying DNA methylation changes, except for CDC20 and ZCCHC24. Other TFs with fewer outgoing links to signature genes showed greatly altered expression levels in PA I in response to strong DNA methylation changes (e.g. EGR3, EN2, EOMES, NR0B1, PAX6, SATB2, ZIC1, ZIC2, ZIC3, ZIC4).

This situation was quite different for AS II, AS III and GBM IV (Fig. 8b). Four central hub TFs showed strongly altered expression levels in response to directly underlying gene copy number mutations (CDC20, GPR123, ZNF365, ZNF488), whereas other hub TFs showed strong underexpression without underlying deletions (e.g. CHD5, HLF, TBR1, THRB, VIP). Again, TFs with altered expression and/or copy number mutations were part of three major functional categories as observed for PA I before. The majority of TFs was involved in development and differentiation (e.g. EGR2, EMX2, DACH2, MEOX2, SOX11). Other TFs were involved in cell cycle control, proliferation, apoptosis and DNA repair (e.g. CCNA1, CDC20, CHD5, TP53, ZNF365). Some TFs were involved in the regulation of chromatin remodeling and DNA methylation (CHD5, EZH2, PAXIP1, ZNF300).

## Conclusions

Our computational study revealed similarities and differences in gene expression levels between astrocytomas of all four WHO grades under consideration of astrocytoma type-specific normal brain references. We compared all four considered astrocytoma grades (PA I, AS II, AS III, GBM IV) at the level of individual genes and cancer-relevant signaling pathways. Thereby, we identified many genes that were exclusively under- or overexpressed in a specific astrocytoma grade. In addition, we also revealed many genes that showed the same pattern of under- or overexpression in specific subsets of astrocytoma grades. We discussed many of these genes in the background of the currently existing literature and we summarized selected astrocytoma type-specific differentially expressed genes that might be of interest for future studies that aim at the development of novel markers. We further observed at the level of individual genes and cancer-relevant signaling pathways that the number of differentially expressed genes typically increased with the astrocytoma grade. This trend suggests an association of transcriptional alterations with the increased tumor aggressiveness of the different astrocytoma grades. Interestingly, the cytokine receptor interaction pathway escaped this general trend. Nearly the same number of overexpressed genes were observed for PA I and GBM IV in this pathway. Detailed studies further identified commonly and exclusively overexpressed genes in the cytokine receptor interaction pathway for PA I and GBM IV and further revealed that only genes that were overexpressed in GBM IV were significantly enriched for known cancer genes involved in aggressiveness, invasion and poor outcome. Moreover, this in-depth analysis also revealed a characteristic expression patterns of CX3CL1 (fractalkine) and its receptor CX3CR1 that distinguished PA I from AS II, AS III and GBM IV. These genes are involved in glioma invasion and progression of malignant astrocytomas [117]. Strong overexpression of both genes in PA I in comparison to higher grade astrocytomas suggests a potential contribution to the non-invasive growth behavior of PA I. Thus, it might be worth to validate this potential link by gene knockdowns in a future study.

Surprisingly, PA I was strongly associated with the mesenchymal subtype, which is typically observed for very aggressive GBM IV. Additional analyses indicated that the tumor micro-environment may have a greater contribution to the manifestation of the mesenchymal subtype than the tumor biology itself, which might explain the seemingly contradiction between the similarity in terms of subtype classification and the very different clinical course of mostly benign PA I and highly malignant GBM IV. In accordance with this, we found that the endothelial cell marker ANGPT2 (alias ANG2) was highly overexpressed in PA I and GBM IV but not in AS II or AS III. Using immunohistochemistry, we confirmed that PA I and GBM IV showed Ang2-positive endothelial cells in regions with activated blood vessels. This feature was largely absent in AS II and AS III. Thus, our study suggests that microvascular proliferation and necrosis, which both have been described as common histological features of PA I and GBM IV [8], contribute at least to some extent to the observation of the mesenchymal subtype.

We also revealed major transcriptional regulators that distinguished PA I from AS II, AS III and GBM IV based on a computationally inferred signature-specific transcriptional regulatory network. We found that many of the differentially expressed central transcriptional regulators play important roles in cell cycle regulation, chromatin remodeling, or brain development and differentiation. Further analyses indicated that the differential expression of transcriptional regulators was mainly driven by directly underlying DNA methylation changes in PA I or gene copy number alterations in AS II, AS III and GBM IV. We note that the impacts of DNA methylation changes on transcriptional regulators in AS II, AS III and GBM IV could not be compared to those in PA I, because DNA methylation profiles were not available for AS II, AS III and GBM IV tumors from Rembrandt. This could be addressed in a future study using DNA methylation profiles measured for AS II, AS III and GBM IV from TCGA brain tumor cohorts.

We are aware that our network approach can also be utilized for the analysis of a molecular signature that distinguishes all four astrocytoma types. However, this should be done based on a larger data set including additional astrocytoma samples from other resources to ensure robustness and transferability. A future study could for example utilize additional publicly available astrocytoma data sets (e.g. TCGA and ICGC data sets and other smaller studies) and further try to directly integrate additional omics layers (e.g. gene copy numbers, DNA methylation profiles, single nucleotide polymorphisms).

Altogether, our study confirmed many known findings and revealed novel interesting insights into astrocytoma biology and therefore represents a valuable resource for future studies.

## Additional files

Additional file 1
**Contains supporting Tables S1–S7.** **Table S1:** Summary of considered astrocytoma samples. **Table S2:** Summary of t-test results for PA I, AS II, AS III and GBM IV. **Table S3:** Signature genes distinguishing PA I from AS II, AS III and GBM IV. **Table S4:** Regulatory network associated with expression differences of signature genes. **Table S5:** Summary of integrated gene annotations. **Table S6:** Verhaak classification results for PA I, AS II, AS III and GBM IV. **Table S7:** Correlation statistics for macrophage marker genes included in the Verhaak-classifier. (ZIP 3287 kb)

Additional file 2
**Contains supporting Texts S1–S5 and supporting Figures S1–S9.** **Text S1:** Processing of Rembrandt gene copy number data. **Text S2:** Lasso-based regulatory network inference. **Text S3:** Endothelial cells express Ang2 in PA I and GBM IV. **Text S4:** Network validation based on independent glioma cohorts. **Text S5:** Comparison of motif search-based and gene expression-based TF-target links. **Figure S1:** Age distribution of PA I, AS II, AS III and GBM IV patients. **Figure S2:** Functional categorization of differentially expressed genes. **Figure S3:** Verhaak classification results of two independent PA I cohorts. **Figure S4:** Ang2 immunohistochemistry. **Figure S5:** Associations of PA I, AS II, AS III and GBM IV with hypermethylator subtype. **Figure S6:** Differential expression in individual signaling pathways. **Figure S7:** Gene expression-based regulatory network validation. **Figure S8:** Overlap of network-based TF-target interactions and motif-based TF-binding sites. **Figure S9:** TF-TF interaction network. (PDF 1249 kb)

## Abbreviations

WHO: World Health Organization
PA I: pilocytic astrocytoma WHO grade I
AS II: diffuse astrocytoma WHO grade II
AS III: anaplastic astrocytoma WHO grade III
GBM IV: glioblastoma WHO grade IV
TF: transcription factor/cofactor

## References

[1] Ohgaki H, Kleihues P (2005). Population-based studies on incidence, survival rates, and genetic alterations in astrocytic and oligodendroglial gliomas. J Neuropathol Exp Neurol.

[2] Canoll P, Goldman JE (2008). The interface between glial progenitors and gliomas. Acta Neuropathol.

[3] Chen J, McKay RM, Parada LF (2012). Malignant Glioma: Lessons from Genomics, Mouse Models, and Stem Cells. Cell.

[4] Louis DN, Ohgaki H, Wiestler OD, Cavenee WK, Burger PC, Jouvet A (2007). WHO classification of tumours of the central nervous system. Acta Neuropathol.

[5] Jones DT, Gronych J, Lichter P, Witt O, Pfister SM (2012). MAPK pathway activation in pilocytic astrocytoma. Cell Mol Life Sci.

[6] Armstrong GT, Conklin HM, Huang S, Srivastava D, Sanford R, Ellison DW (2011). Survival and long-term health and cognitive outcomes after low-grade glioma. Neuro Oncol.

[7] Jones DT, Hutter B, Jäger N, Korshunov A, Kool M, Warnatz HJ (2013). Recurrent somatic alterations of FGFR1 and NTRK2 in pilocytic astrocytoma. Nat Genet.

[8] Kurwale NS, Suri V, Suri A, Sarkar C, Gupta DK, Sharma BS (2011). Predictive factors for early symptomatic recurrence in pilocytic astrocytoma: does angiogenesis have a role to play?. J Clin Neurosci.

[9] Rodriguez EF, Scheithauer BW, Giannini C, Rynearson A, Cen L, Hoesley B (2011). PI3K/AKT pathway alterations are associated with clinically aggressive and histologically anaplastic subsets of pilocytic astrocytoma. Acta Neuropathol.

[10] Tonn JC, Westphal M, Rutka JT, Grossman SA (2005). Neuro-oncology of CNS tumors. ISBN: 978-3540258339.

[11] Kelly PJ (2010). Gliomas: Survival, origin and early detection. Surg Neurol Int.

[12] Ohgaki H, Kleihues P (2009). Genetic alterations and signaling pathways in the evolution of gliomas. Cancer Sci.

[13] Ohgaki H, Kleihues P (2013). The definition of primary and secondary glioblastoma. Clin Cancer Res.

[14] Johnson BE, Mazor T, Hong C, Barnes M, Aihara K, McLean CY (2014). Mutational analysis reveals the origin and therapy-driven evolution of recurrent glioma. Science.

[15] Tove LL, Hansson HA, Stein S, Sverre HT (2012). Prognostic value of histological features in diffuse astrocytomas WHO grade II. Int J Clin Exp Pathol.

[16] Smoll NR, Hamilton B (2014). Incidence and relative survival of anaplastic astrocytomas. Neuro Oncol.

[17] Nuno M, Birch K, Mukherjee D, Sarmiento JM, Black KL, Patil CG (2013). Survival and prognostic factors of anaplastic gliomas. Neurosurgery.

[18] Sturm D, Bender S, Jones DT, Lichter P, Grill J, Becher O (2014). Paediatric and adult glioblastoma: multiforme (epi)genomic culprits emerge. Nat Rev Cancer.

[19] Parsons DW, Jones S, Zhang X, Lin JC, Leary RJ, Angenendt P (2008). An integrated genomic analysis of human glioblastoma multiforme. Science.

[20] Gorovets D, Kannan K, Shen R, Kastenhuber ER, Islamdoust N, Campos C (2012). IDH mutation and neuroglial developmental features define clinically distinct subclasses of lower grade diffuse astrocytic glioma. Clin Cancer Res.

[21] Stupp R, Mason WP, van den Bent MJ, Weller M, Fisher B, Taphoorn MJ (2005). Radiotherapy plus concomitant and adjuvant temozolomide for glioblastoma. N Engl J Med.

[22] Taveras JM, Thompson HG, Pool JL (1962). Should we treat glioblastoma multiforme? A study of survival in 425 cases. Am J Roentgenol Radium Ther Nucl Med.

[23] The Cancer Genome Atlas Nework (2008). Comprehensive genomic characterization defines human glioblastoma genes and core pathways. Nature.

[24] Brennan CW, Verhaak RG, McKenna A, Campos B, Noushmehr H, Salama SR (2013). The somatic genomic landscape of glioblastoma. Cell.

[25] Verhaak RG, Hoadley KA, Purdom E, Wang V, Qi Y, Wilkerson MD (2010). Integrated genomic analysis identifies clinically relevant subtypes of glioblastoma characterized by abnormalities in PDGFRA, IDH1, EGFR, and NF1. Cancer Cell.

[26] Noushmehr H, Weisenberger DJ, Diefes K, Phillips HS, Pujara K, Berman BP (2010). Identification of a CpG island methylator phenotype that defines a distinct subgroup of glioma. Cancer Cell.

[27] Cooper LAD, Gutman DA, Long Q, Johnson BA, Cholleti SR, Kurc T (2010). The Proneural Molecular Signature Is Enriched in Oligodendrogliomas and Predicts Improved Survival among Diffuse Gliomas. PLoS One.

[28] Seifert M, Abou-El-Ardat K, Friedrich B, Klink B, Deutsch A (2014). Autoregressive Higher-Order Hidden Markov Models: Exploiting Local Chromosomal Dependencies in the Analysis of Tumor Expression Profiles. PLoS One.

[29] Carro MS, Lim WK, Alvarez MJ, Bollo RJ, Zhao X, Snyder EY (2010). The transcriptional network for mesenchymal transformation of brain tumours. Nature.

[30] Jörnsten R, Abenius T, Kling T, Schmidt L, Johansson E, Nordling TE (2011). Network modeling of the transcriptional effects of copy number aberrations in glioblastoma. Mol Syst Biol..

[31] Deshmukh H, Yu J, Shaik J, MacDonald TJ, Perry A, Payton JE (2011). Identification of transcriptional regulatory networks specific to pilocytic astrocytoma. BMC Med Genomics..

[32] Setty M, Helmy K, Khan AA, Silber J, Arvey A, Neezen F (2012). Inferring transcriptional and microRNA-mediated regulatory programs in glioblastoma. Mol Syst Biol..

[33] Wang C, Funk CC, Eddy JA, Price ND (2013). Transcriptional analysis of aggressiveness and heterogeneity across grades of astrocytomas. PLoS One.

[34] Huang H, Hara A, Homma T, Yonekawa Y, Ohgaki H (2005). Altered expression of immune defense genes in pilocytic astrocytomas. J Neuropathol Exp Neurol..

[35] Rorive S, Maris C, Debeir O, Sandras F, Vidaud M, Bièche I (2006). Exploring the distinctive biological characteristics of pilocytic and low-grade diffuse astrocytomas using microarray gene expression profiles. J Neuropathol Exp Neurol.

[36] Rickman DS, Bobek MP, Misek DE, Kuick R, Blaivas M, Kurnit DM (2001). Distinctive molecular profiles of high-grade and low-grade gliomas based on oligonucleotide microarray analysis. Cancer Res.

[37] Hunter S, Young A, Olson J, Brat DJ, Bowers G, Wilcox JN (2002). Differential expression between pilocytic and anaplastic astrocytomas: identification of apolipoprotein D as a marker for low-grade, non-infiltrating primary CNS neoplasms. J Neuropathol Exp Neurol.

[38] Lambert SR, Witt H, Hovestadt V, Zucknick M, Kool M, Pearson DM (2013). Differential expression and methylation of brain developmental genes define location-specific subsets of pilocytic astrocytoma. Acta Neuropathol.

[39] Wu Z, Irizarry RA, Gentleman R, Martinez-Murillo F, Spencer F (2004). A model-based background adjustment for oligonucleotide expression arrays. J Am Statist Assoc.

[40] Madhavan S, Zenklusen JC, Kotliarov Y, Sahmi H, Fine HA, Buetow K (2009). Rembrandt: Helping personalized medicine become a reality through integrative translational research. Mol. Cancer Res.

[41] Sun L, Hui AM, Su Q, Vortmeyer A, Kotliarov Y, Pastorino S (2006). Neuronal and glioma-derived stem cell factor induces angiogenesis within the brain. Cancer Cell.

[42] Benjamini Y, Hochberg Y (1995). Controlling the false discovery rate: a practical and powerful approach to multiple testing. J R Stat Soc Series B.

[43] Tibshirani R (1996). Regression shrinkage and selection via the lasso. J R Stat Soc Series B.

[44] Lockhart R, Taylor J, Tibshirani RJ, Tibshirani R (2014). A significance test for the lasso. Ann Stat.

[45] Futreal PA, Coin L, Marshall M, Down T, Hubbard T, Wooster R (2004). A census of human cancer genes. Nar Rev Cancer.

[46] Sharma MK, Mansur DB, Reifenberger G, Perry A, Leonard JR, Aldape KD (2007). Distinct genetic signatures among pilocytic astrocytomas relate to their brain region origin. Cancer Res.

[47] Klein R, Roggendorf W (2001). Increased microglia proliferation separates pilocytic astrocytomas from diffuse astrocytomas: a double labeling study. Acta Neuropathol.

[48] Herder V, Iskandar CD, Kegler K, Hansmann F, Elmarabet SA, Khan MA, et al. Dynamic changes of microglia/macrophage M1 and M2 polarization in Theiler’s murine encephalomyelitis. Brain Pathol. 2015:1750–3639. doi:10.1111/bpa.12238. 10.1111/bpa.12238 PMC8029129 25495532

[49] Stratmann A, Risau W, Plate KH (1998). Cell type-specific expression of angiopoietin-1 and angiopoietin-2 suggests a role in glioblastoma angiogenesis. Am J Pathol.

[50] Turcan S (2012). IDH1 mutation is sufficient to establish the glioma hypermethylator phenotype. Nature.

[51] Eden E, Navon R, Steinfeld I, Lipson D, Yakhini Z (2009). GOrilla: A tool for discovery and visualization of enriched GO terms in ranked gene lists. BMC Bioinformatics..

[52] Baala L, Briault S, Etchevers HC, Laumonnier F, Natiq A, Amiel J (2007). Homozygous silencing of T-box transcription factor EOMES leads to microcephaly with polymicrogyria and corpus callosum agenesis. Nat Genet.

[53] Reuss DE, Sahm F, Schrimpf D, Wiestler B, Capper D, Koelsche C (2015). ATRX and IDH1-R132H immunohistochemistry with subsequent copy number analysis and IDH sequencing as a basis for an integrated? diagnostic approach for adult astrocytoma, oligodendroglioma and glioblastoma. Acta Neuropathol.

[54] Fuxe J, Akusjärvi G, Goike HM, Roos G, Collins VP, Pettersson RF (2000). Adenovirus-mediated overexpression of p15INK4B inhibits human glioma cell growth, induces replicative senescence, and inhibits telomerase activity similarly to p16INK4A. Cell Growth Differ.

[55] Otero JJ, Rowitch D, Vandenberg S (2011). OLIG2 is differentially expressed in pediatric astrocytic and in ependymal neoplasms. J Neurooncol.

[56] Wadhwa S, Nag TC, Jindal A, Kushwaha R, Mahapatra AK, Sarkar C (2003). Expression of the neurotrophin receptors Trk A and Trk B in adult human astrocytoma and glioblastoma. J Biosci.

[57] Murat A, Migliavacca E, Gorlia T, Lambiv WL, Shay T, Hamou MF (2008). Stem cell-related self-renewal signature and high epidermal growth factor receptor expression associated with resistance to concomitant chemoradiotherapy in glioblastoma. J Clin Oncol.

[58] Li F, Jang H, Puttabyatappa M, Jo EJJM Curry (2012). Ovarian FAM110C (Family with Sequence Similarity 110C): Induction during the periovulatory period and regulation of granulosa cell cycle kinetics in rats. Biol Reprod.

[59] Miotto E, Sabbioni S, Veronese A, Calin GA, Gullini S, Liboni A (2004). Frequent aberrant methylation of the CDH4 gene promoter in human colorectal and gastric cancer. Cancer Res.

[60] Xie Q, Flavahan WA, Bao S, Rich J (2014). The tailless root of glioma: Cancer stem cells. Cell Stem Cell.

[61] Phillips HS, Karbanda S, Chen R, Forrest WF, Soriano RH, Wu TD (2006). Molecular subclasses of high-grade glioma predict prognosis, delineate a pattern of disease progression, and resemble stages in neurogenesis. Cancer Cell.

[62] Auvergne RM, Sim FJ, Wang S, Chandler-Militello D, Burch J, Al Fanek Y (2013). Transcriptional differences between normal and glioma-derived glial progenitor cells identify a core set of dysregulated genes. Cel Rep.

[63] Zhang L, Chen LH, Wan H, Yang R, Wang Z, Feng J (2014). Exome sequencing identifies somatic gain-of-function PPM1D mutations in brainstem gliomas. Nat Genet.

[64] Wang P, Rao J, Yang H, Zhao H, Yang L (2011). PPM1D silencing by lentiviral-mediated RNA interference inhibits proliferation and invasion of human glioma cells. J Huazhong Univ Sci Technolog Med Sci.

[65] Park KH, Choi SE, Eom M, Kang Y (2005). Downregulation of the anaphase-promoting complex (APC)7 in invasive ductal carcinomas of the breast and its clinicopathologic relationships. Breast Cancer Res.

[66] Chen Y, Cai J, Murphy TJ, Jones DP (2002). Overexpressed human mitochondrial thioredoxin confers resistance to oxidant-induced apoptosis in human osteosarcoma cells. J Biol Chem.

[67] Harper J, Yan L, Loureiro RM, Wu I, Fang J, D’Amore PA (2007). Repression of vascular endothelial growth factor expression by the zinc finger transcription factor ZNF24. Cancer Res.

[68] Hatanpaa KJ, Burma S, Zhao D, Habib AA (2010). Epidermal growth factor receptor in glioma: signal transduction, neuropathology, imaging, and radioresistance. Neoplasia.

[69] Guo P, Hu B, Gu W, Xu L, Wang D, Huang HJ (2003). Platelet-derived growth factor-B enhances glioma angiogenesis by stimulating vascular endothelial growth factor expression in tumor endothelia and by promoting pericyte recruitment. Am J Pathol.

[70] Nazarenko I, Hede SM, He X, Hedrén A, Thompson J, Lindström MS (2012). PDGF and PDGF receptors in glioma. Ups J Med Sci.

[71] Riemenschneider MJ, Büschges R, Wolter M, Reifenberger J, Boström J, Kraus JA (1999). Amplification and overexpression of the MDM4 (MDMX) gene from 1q32 in a subset of malignant gliomas without TP53 mutation or MDM2 amplification. Cancer Res.

[72] Riemenschneider MJ, Knobbe CB, Reifenberger G (2003). Refined mapping of 1q32 amplicons in malignant gliomas confirms MDM4 as the main amplification target. Int J Cancer.

[73] Pu P, Kang C, Li J, Wang G (2005). Suppression of glioma-cell survival by antisense and dominant-negative AKT2 RNA. Cancer Biol Med.

[74] Zhang J, Han L, Zhang A, Wang Y, Yue X, You Y (2010). AKT2 expression is associated with glioma malignant progression and required for cell survival and invasion. Oncol Rep.

[75] Morrision RS, Yamaguchi F, Bruner JM, Tang M, McKeehan W, Berger MS (1994). Fibroblast growth factor receptor gene expression and immunoreactivity are elevated in human glioblastoma multiforme. Cancer Res.

[76] Bai J, Mei PJ, Liu H, Li C, Li W, Wu YP (2012). BRG1 expression is increased in human glioma and controls glioma cell proliferation, migration and invasion in vitro. J Cancer Res Clin Oncol.

[77] Yan H, Yang K, Xiao H, Zou YJ, Zhang WB, Liu HY (2012). Over-expression of cofilin-1 and phosphoglycerate kinase 1 in astrocytomas involved in pathogenesis of radioresistance. CNS Neurosci Ther.

[78] Ding H, Cheng YJ, Yan H, Zhang R, Zhao JB, Qian CF (2014). Phosphoglycerate kinase 1 promotes radioresistance in U251 human glioma cells. Oncol Rep.

[79] Phung TL, Du W, Xue Q, Ayyaswamy S, Gerald D, Antonello Z (2015). Akt1 and Akt3 exert opposing roles in the regulation of vascular tumor growth. Cancer Res.

[80] Toedt G, Barbus S, Wolter M, Felsberg J, Tews B, Blond F (2011). Molecular signatures classify astrocytic gliomas by IDH1 mutation status. Int J Cancer.

[81] Palani M, Arunkumar R, Vanisree AJ (2014). Methylation and expression patterns of tropomyosin-related kinase genes in different grades of glioma. Neuromolecular Med.

[82] Xu Y, Stamenkovic I, Yu Q (2010). CD44 attenuates activation of the hippo signaling pathway and is a prime therapeutic target for glioblastoma. Cancer Res.

[83] Li Z, Bao S, Wu Q, Wang H, Eyler C, Sathornsumetee S (2009). Hypoxia-inducible factors regulate tumorigenic capacity of glioma stem cells. Cancer Cell.

[84] Ludwig S, Engel K, Hoffmeyer A, Sithanandam G, Neufeld B, Palm D (1996). 3pK, a novel mitogen-activated protein (MAP) kinase-activated protein kinase, is targeted by three MAP kinase pathways. Mol Cell Biol.

[85] Nakada M, Kita D, Watanabe T, Hayashi Y, Teng L, Pyko IV (2011). Aberrant signaling pathways in glioma. Cancer.

[86] Woitach JT, Zhang M, Niu CH, Thorgeirsson SS (1998). A retinoblastoma-binding protein that affects cell-cycle control and confers transforming ability. Nat Genet.

[87] Zou J, Wang K, Han L, Zhang A, Shi Z, Pu P (2011). AKT1 and AKT2 promote malignant transformation in human brain glioma LN229 cells. Clin Oncol Cancer Res.

[88] Orian JM, Vasilopoulos K, Yoshida S, Kaye AH, Chow CW, Gonzales MF (1992). Overexpression of multiple oncogenes related to histological grade of astrocytic glioma. Br J Cancer.

[89] Nagpal J, Jamoona A, Gulati ND, Mohan A, Braun A, Murdi R (2006). Revisiting the role of p53 in primary and secondary glioblastomas. Anticancer Res.

[90] Pollack IF, Hamilton RL, Finkelstein SD, Campbell JW, Martinez AJ, Sherwin RN (1997). The relationship between TP53 mutations and overexpression of p53 and prognosis in malignant gliomas of childhood. Cancer Res.

[91] Holland EC, Hively WP, Gallo V, Varmus HE (1998). Modeling mutations in the G1 arrest pathway in human gliomas: overexpression of CDK4 but not loss of INK4aARF induces hyperploidy in cultured mouse astrocytes. Genes Dev.

[92] Huang ZY, Baldwin RL, Hedrick NM, Gutmann DH (2002). Astrocyte-specific expression of CDK4 is not sufficient for tumor formation, but cooperates with p53 heterozygosity to provide a growth advantage for astrocytes in vivo. Oncogene.

[93] Lyustikman Y, Momota H, Pao W, Holland EC (2008). Constitutive activation of Raf-1 induces glioma formation in mice. Neoplasia.

[94] Wang JB, Dong DF, Wang MD, Gao K (2014). IDH1 overexpression induced chemotherapy resistance and IDH1 mutation enhanced chemotherapy sensitivity in glioma cells in vitro and in vivo. Asian Pac J Cancer Prev.

[95] Ahn YH, Yang Y, Gibbons DL, Creighton CJ, Yang F, Wistuba II (2011). Map2k4 functions as a tumor suppressor in lung adenocarcinoma and inhibits tumor cell invasion by decreasing peroxisome proliferator-activated receptor *γ*2 expression. Mol Biol Cell.

[96] Lee EW, Lee MS, Camus S, Ghim J, Yang MR, Oh W (2009). Differential regulation of p53 and p21 by MKRN1 E3 ligase controls cell cycle arrest and apoptosis. EMBO Journal.

[97] Wang E, Zhang C, Polavaram N, Liu F, Wu G, Schroeder MA, et al. The role of factor inhibiting HIF (FIH-1) in inhibiting HIF-1 transcriptional activity in glioblastoma multiforme. PLoS One.2014;9(e86102). doi:10.1371/journal.pone.0086102. 10.1371/journal.pone.0086102 PMC3900478 24465898

[98] Viré E, Brenner C, Deplus R, Blanchon L, Fraga M, Didelot C (2006). The Polycomb group protein EZH2 directly controls DNA methylation. Nature.

[99] Schlesinger Y, Straussman R, Keshet I, Farkash S, Hecht M, Zimmerman J (2007). Polycomb-mediated methylation on Lys27 of histone H3 pre-marks genes for de novo methylation in cancer. Nat Genet.

[100] Toda M (2013). Glioma stem cells and immunotherapy for the treatment of malignant gliomas. ISRN Oncology.

[101] Xie Q, Wu Q, Mack S, Yang K, Kim L, Hubert C (2015). CDC20 maintains tumor initiating cells. Oncotarget..

[102] Annovazzi L, Mellai M, Caldera V, Valente G, Schiffer D (2011). SOX2 expression and amplification in gliomas and glioma cell lines. Cancer Genomics Proteomics.

[103] Berezovsky AD, Poisson LM, Cherba D, Webb CP, Transou AD, Lemke NW (2014). Sox2 promotes malignancy in glioblastoma by regulating plasticity and astrocytic differentiation. Neoplasia.

[104] Ducan CG, Killela PJ, Payne CA, Lampson B, Chen WC, Liu J (2010). Integrated genomic analyses identify ERRFI1 and TACC3 as glioblastoma-targeted genes. Oncotarget.

[105] Parker BC, Annala MJ, Cogdell DE, Granberg KJ, Sun Y, Ji P (2013). The tumorigenic FGFR3-TACC3 gene fusion escapes mir-99a regulation in glioblastoma. J Clin Invest.

[106] Li S, Chou AP, Chen W, Chen R, Deng Y, Phillips HS (2013). Overexpression of isocitrate dehydrogenase mutant proteins renders glioma cells more sensitive to radiation. Neuro Oncol.

[107] Mantovani A, Allavena P, Sica A, Balkwill F (2008). Cancer-related inflammation. Nature.

[108] Allavena P, Germano G, Marchesi F, Mantovani A (2011). Chemokines in cancer related inflammation. Exp Cell Res.

[109] Guven-Maiorov E, Acuner-Ozbabacan SE, Keskin O, Gursoy A, Nussinov R (2014). Structural pathways of cytokines may illuminate their roles in regulation of cancer development and immunotherapy. Cancers (Basel).

[110] Ilyin SE, González-Gómez I, Gilles FH, Plata-Salamán CR (1998). Interleukin-1 alpha (IL-1 alpha), IL-1 beta, IL-1 receptor type I, IL-1 receptor antagonist, and TGF-beta 1 mRNAs in pediatric astrocytomas, ependymomas, and primitive neuroectodermal tumors. Mol Chem Neuropathol.

[111] Sasaki A, Ishiuchi S, Kanda T, Hasegawa M, Nakazato Y (2001). Analysis of interleukin-6 gene expression in primary human gliomas, glioblastoma xenografts, and glioblastoma cell lines. Brain Tumor Pathol.

[112] Plata-Salamán CR (2002). Brain cytokines and disease. Acta Neuropsychiatrica.

[113] Zhou Y, Larsen PH, Hao C, Yong VW (2002). CXCR4 is a major chemokine receptor on glioma cells and mediates their survival. J Biol Chem.

[114] Kouno J, Nagai H, Nagahata T, Onda M, Yamaguchi H, Adachi K (2004). Up-regulation of CC chemokine, CCL3L1, and receptors, CCR3, CCR5 in human glioblastoma that promotes cell growth. J Neurooncol.

[115] Ludwig A, Schulte A, Schnack C, Hundhausen C, Reiss K, Brodway N (2005). Enhanced expression and shedding of the transmembrane chemokine CXCL16 by reactive astrocytes and glioma cells. J Neurochem.

[116] Sciumé G, Soriani A, Piccoli M, Frati L, Santoni A, Bernardini G (2010). CX3CR1/CX3CL1 axis negatively controls glioma cell invasion and is modulated by transforming growth factor–1. Neuro Oncol.

[117] Yao X, Liu Y, Huang J, Zhou Y, Chen K, Gong W, et al. The role of chemoattractant receptors in the progression of glioma, Glioma - Exploring its biology and practical relevance. InTech, Anirban Ghosh (Ed.) 2011. doi:10.5772/22154.

[118] Zhou J, Xiang Y, Yoshimura T, Chen K, Gong W, Huang J (2014). The role of chemoattractant receptors in shaping the tumor microenvironment. Biomed Res Int..

[119] Talasila KM, Soentgerath A, Euskirchen P, Rosland GV, Wang J, Huszthy PC (2013). EGFR wild-type amplification and activation promote invasion and development of glioblastoma independent of angiogenesis. Acta Neuropathol.

[120] Gong J, Zhu S, Zhang Y, Wang J (2014). Interplay of VEGFa and MMP2 regulates invasion of glioblastoma. Tumour Biol.

[121] Bazan JF, Bacon KB, Hardiman G, Wang W, Soo K, Rossi D (1997). A new class of membrane-bound chemokine with a CX3C motif. Nature.

[122] Imai T, Hieshima K, Haskell C, Baba M, Nagira M, Nishimura M (1997). Identification and molecular characterization of fractalkine receptor CX3CR1, which mediates both leukocyte migration and adhesion. Cell.

[123] Marchesi F, Locatelli M, Solinas G, Erreni M, Allavena P, Mantovani A (2010). Role of CX3CR1/CX3CL1 axis in primary and secondary involvement of the nervous system by cancer. J Neuroimmunol.

[124] Lauro C, Catalano M, Trettel F, Mainiero F, Ciotti MT, Eusebi F (2006). The chemokine CX3CL1 reduces migration and increases adhesion of neurons with mechanisms dependent on the beta1 integrin subunit. J Immunol.

[125] Claes A, Idema AJ, Wesseling P (2007). Diffuse glioma growth: a guerilla war. Acta Neuropathol.

[126] Cheng Y, Pang JC, Ng HK, Ding M, Zhang SF, Zheng J (2000). Pilocytic astrocytomas do not show most of the genetic changes commonly seen in diffuse astrocytomas. Histopathology.

[127] Yang L, Li N, Wang C, Yu Y, Yuan L, Zhang M (2004). Cyclin L2, a novel RNA polymerase II-associated cyclin, is involved in pre-mRNA splicing and induces apoptosis of human hepatocellular carcinoma cells. J Biol Chem.

[128] Ley TJ, Mardis ER, Ding L, Fulton B, McLellan MD, Chen K (2008). DNA sequencing of a cytogenetically normal acute myeloid leukaemia genome. Nature.

[129] Bulfone A, Smiga SM, Shimamura K, Peterson A, Puelles L, Rubenstein JL (1995). T-brain-1: a homolog of Brachyury whose expression defines molecularly distinct domains within the cerebral cortex. Neuron.

[130] Kanehisa M, Goto S (2000). KEGG: kyoto encyclopedia of genes and genomes. Nucl Acids Res.

