# Supplementary material for: Comparative transcriptomics reveals similarities and differences between astrocytoma grades
Source: BMC Cancer. 2015 Dec 16;15:952. doi: 10.1186/s12885-015-1939-9 (PMC4682229; doi:10.1186/s12885-015-1939-9)
Supplement: Additional file 2 — Contains supporting Texts S1–S5 and supporting Figures S1–S9. Text S1: Processing of Rembrandt gene copy number data. Text S2: Lasso-based regulatory network inference. Text S3: Endothelial cells express Ang2 in PA I and GBM IV. Text S4: Network validation based on independent glioma cohorts. Text S5: Comparison of motif search-based and gene expression-based TF-target links. Figure S1: Age distribution of PA I, AS II, AS III and GBM IV patients. Figure S2: Functional categorization of differentially expressed genes. Figure S3: Verhaak classification results of two independent PA I cohorts. Figure S4: Ang2 immunohistochemistry. Figure S5: Associations of PA I, AS II, AS III and GBM IV with hypermethylator subtype. Figure S6: Differential expression in individual signaling pathways. Figure S7: Gene expression-based regulatory network validation. Figure S8: Overlap of network-based TF-target interactions and motif-based TF-binding sites. Figure S9: TF-TF interaction network. (PDF 1249 kb) [file 12885_2015_1939_MOESM2_ESM.pdf]

Comparative Transcriptomics Reveals Similarities and Differences between Astrocytoma Grades

Supplementary Texts and Figures

Michael Seifert[1,2,5], Martin Garbe[1], Betty Friedrich[1,3],
Michel Mittelbronn[4] and Barbara Klink[5,6,7]

[1]Innovative Methods of Computing, Center for Information Services and High Performance Computing, Dresden University of Technology, Dresden, Germany; [2]Cellular Networks and Systems Biology, University of Cologne, CECAD, Cologne, Germany; [3]Institute of Molecular Systems Biology, Zurich, Switzerland; [4]Institute of Neurology (Edinger Institute), Goethe University, Frankfurt, Germany; [5]Institute for Clinical Genetics, Faculty of Medicine Carl Gustav Carus, Dresden University of Technology, Dresden, Germany; [6]German Cancer Consortium (DKTK), Dresden, Germany; [7]German Cancer Research Center (DKFZ), Heidelberg, Germany

Contact: michael.seifert@tu-dresden.de

Contents

1 Supplementary Texts 2
- 1.1 Text S1: Processing of Rembrandt copy number data . . . 2
- 1.2 Text S2: Lasso-based regulatory network inference . . . 2
- 1.3 Text S3: Endothelial cells express Ang2 in PA I and GBM IV . . . 3
  - 1.3.1 Immunohistochemistry . . . 3
  - 1.3.2 Endothelial cells are the source of Ang2 protein expression in both PA I and GBM IV 3
- 1.4 Text S4: Network validation based on independent glioma cohorts . . . 4
  - 1.4.1 Independent glioma gene expression data for regulatory network validation . . . 4
  - 1.4.2 Network validation using independent glioma cohorts . . . 4
- 1.5 Text S5: Comparison of motif search-based and gene expression-based TF-target links . 4
  - 1.5.1 Motif search-based TF-target identification . . . 4
  - 1.5.2 Signature-specific TF-target validation . . . 5

2 Supplementary Figures 6
- 2.1 Figure S1: Age distribution of patients diagnosed with astrocytomas of different WHO grades . . . 6
- 2.2 Figure S2: Functional categorization of differentially expressed genes . . . 6
- 2.3 Figure S3: Verhaak-subtype classification of two independent PA I cohorts . . . 7
- 2.4 Figure S4: Ang2 expression in endothelial cells in PA I and GBM IV tumors . . . 7
- 2.5 Figure S5: Associations of astrocytomas with hypermethylator subtype . . . 8
- 2.6 Figure S6: Differential expression in individual signaling pathways . . . 9
- 2.7 Figure S7: Validation of the regulatory network inferred from the molecular signature distinguishing PA I from AS II, AS III and GBM IV . . . 10
- 2.8 Figure S8: Overlap of network-based TF-target gene interactions and motif-based TF-binding sites . . . 11
- 2.9 Figure S9: TF-TF-interaction network distinguishing PA I from AS II, AS III and GBM IV . 12

1 Supplementary Texts

1.1 Text S1: Processing of Rembrandt copy number data

We downloaded Affymetrix 100K SNP array data from Rembrandt (release 1.5.9) [1], reconstructed hybridization images and performed stringent quality controls to remove all arrays with hybridization artifacts. We identified 78 tumor samples (16 AS II, 17 AS III, 45 GBM IV) and 8 blood reference samples that passed this quality check (Tab. S1). Next, we analyzed these data using R connected to a local installation of the GenePattern server [2]. The SNPFileCreator module was used to transform each array into a single-nucleotide polymorphism (SNP) file in combination with standard dChip normalization of each array against a global blood reference sample (Rembrandt-IDs: E09419 for Xba240 arrays and E10615 for Hind240 arrays). The obtained SNP files of tumor samples were further converted into $\log_2$-ratio copy number profiles under consideration of average blood sample reference measurements. All resulting tumor-specific copy number profiles were further segmented using circular binary segmentation (CBS) [3] to identify individual deletions and amplifications of chromosomal regions. We finally utilized the obtained segmentation profiles to determine corresponding gene copy number measurements for each individual gene in each individual tumor. In some cases, breakpoints occurred within genes and we then utilized the corresponding average log-ratio of involved segments to specify the copy of the affected gene.

1.2 Text S2: Lasso-based regulatory network inference

We considered gene-specific sub-network inference problems to learn a transcriptional regulatory network associated with the expression of molecular signature genes distinguishing PA I from AS II, AS III and GBM IV. Therefore, we focused on the expression levels of $N = 1,089$ signature genes in our data set of in total $D = 125$ astrocytoma samples comprising 47 PA I, 16 AS II, 17 AS III and 45 GBM IV tumors. For each signature gene $i \in \{1, \ldots, N\}$, we assumed that its expression level e_{id} in an astrocytoma $d \in \{1, \ldots, D\}$ can be predicted by a linear combination

$$e_{id} = \sum_{j \in \mathtt{TF} \setminus \{i\}} a_{ji} \cdot e_{jd} \tag{1}$$

of the expression levels e_{jd} of transcriptional regulators $j \in \mathtt{TF} \setminus \{i\}$ that were part of the molecular signature that distinguishes PA I from AS II, AS III and GBM IV. Here, $\mathtt{TF}$ defines the subset of genes in the molecular signature that were annotated as TFs (151 of 1,089). The expression level e_{id} of each gene i in an astrocytoma d is given by the $\log_2$-ratio of the expression level of gene i in astrocytoma d in comparison to the expression level of gene i in the corresponding average normal brain reference. The unknown parameters of this signature gene-specific linear model are given by $\vec{a}_i := (a_{ji})_{j \in \mathtt{TF} \setminus \{i\}}$. Each individual parameter $a_{ji} \in \mathbb{R}$ quantifies the impact of the expression level of transcriptional regulator j on the expression level of signature gene i: (i) $a_{ji} < 0$ specifies that TF j is a putative inhibitor of gene i, (ii) $a_{ji} > 0$ defines that TF j is a putative activator of gene i, and (iii) $a_{ji} = 0$ means that no dependency between j and i exists.

We used lasso (least absolute shrinkage and selection operator) regression [4] to determine a sparse solution for the signature gene-specific linear model in Eq. (1). Lasso minimizes the residual sum of squares

$$\vec{a}_i^* = \operatorname*{argmin}_{\vec{a}_i} \sum_{d=1}^{D} \left(e_{id} - \left(\sum_{j \in \mathtt{TF} \setminus \{i\}} a_{ji} \cdot e_{jd} \right) \right)^2 + \lambda_i \sum_{j \in \mathtt{TF} \setminus \{i\}} |a_{ji}| \tag{2}$$

of the measured expression e_{id} of gene i and the model-based predicted expression of gene i under

consideration of all astrocytomas in dependency of a fixed signature gene-specific complexity parameter λ_i. The complexity parameter λ_i determines the amount of shrinkage of the individual model parameters a_{ji} toward zero, where larger values of λ_i lead to greater shrinkage. This allows to select relevant transcriptional regulators, because irrelevant model parameters will be shrunken to zero. We used the R package glmnet [5] to determine an optimal signature gene-specific complexity parameter and corresponding optimal model parameters. We determined λ_i by averaging the optimal complexity parameters (cv.glmnet: lambda.min) obtained from ten independent repeats of a ten-fold cross-validation across all astrocytomas. We then used this gene-specific complexity parameter λ_i to compute the corresponding optimal model parameters considering all astrocytomas. We further determined the significance of model parameters when they first enter the lasso model in Eq. (2) using a recently developed significance test for lasso [6]. This provides an efficient way to get p-values instead of using computationally expensive permutation strategies. To realize this, we first computed the lasso solution paths for the active transcriptional regulators (model parameters in $\vec{a}_i^*$ that are unequal zero) with respect to all astrocytomas using the R package lars [7]. These results were further evaluated using the R package covTest [8] to obtain p-values that characterize the importance of individual active transcriptional regulators for the signature gene-specific linear model.

We applied this approach to each signature gene to identify corresponding putative transcriptional regulators that best predict the expression levels of the signature gene across all types of astrocytomas. For each signature gene, we only considered potential transcriptional regulators with p-values less than 5×10^{-5} (standard numerical precision limit of covTest). The obtained regulatory network is provided in Tab. S4. Since we used expression data to learn this model, the expression levels of a selected regulator are either positively (potential activator link from regulator to signature gene) or negatively (potential repressor link from regulator to signature gene) correlated with those of the signature gene. Thus, this does not necessarily imply that a selected regulator directly regulates the signature gene via physical interactions. A selected regulator may only have an indirect regulatory impact on the signature gene or may only be co-expressed with the signature gene.

We further validated the predictive power of the obtained regulatory network on independent astrocytoma data sets (Text S4, Fig. S7) and we also evaluated the putative proportion of included direct TF-target gene interactions (Text S5, Fig. S8). All these validation studies clearly indicated that the obtained regulatory network included relevant regulator information to predict the expression levels of signature genes based on the expression profiles of TFs.

1.3 Text S3: Endothelial cells express Ang2 in PA I and GBM IV

1.3.1 Immunohistochemistry

Glioma and control specimens were fixed in 10% formaldehyde and embedded in paraffin. Sections (4µm) were deparaffinized and antigen retrieval was performed. A Ventana Benchmark automated staining system was used for immunohistochemistry of Ang2 (anti-human Ang-2 antibody ((MAB 0983), 1:100 from R&D Biosciences, Minneapolis, MN, USA). For antigen retrieval, cell conditioning 1 (prediluted, Ventana Medical Systems) was used. After incubation with the primary and corresponding secondary antibody, the signal was detected with the Chromo DAB Map kit (Ventana Medical Systems) and with the IHC Ultra Map AP (Ventana Medical Systems) kit. Note that the Ang2 protein is encoded by the ANGPT2 gene (alias ANG2).

1.3.2 Endothelial cells are the source of Ang2 protein expression in both PA I and GBM IV

To assess the cellular source of genes significantly contributing to the clustering of PA I within the mesenchymal subgroup of glioblastoma, we exemplarily analyzed normal and neoplastic CNS tissue for

Ang2 by immunohistochemistry (Fig. S4). Of note, normal white (Fig. S4A) and gray (Fig. S4B) matter were completely devoid of Ang2. In contrast, both PA I (Fig. S4C) and GBM IV (Fig. S4F) showed prominent Ang2-positive endothelial cells in case that activated blood vessel were present, a feature that was largely absent in AS II (Fig. S4D) and AS III (Fig. S4E). This finding indicates that the significantly higher Angpt2 mRNA expression found in PA I and GBM IV is strongly related to the presence of vascular proliferation which - per definition - are only seen in those tumor entities while being absent in WHO grade II and III astrocytomas.

1.4 Text S4: Network validation based on independent glioma cohorts

1.4.1 Independent glioma gene expression data for regulatory network validation

We downloaded raw PA I expression data published in [9] from Gene Expression Omnibus (GSE5675) and normalized all microarrays using GCRMA [10]. This independent PA I cohort contained expression levels of 16,973 genes for 41 PA I tumor samples. We further downloaded processed gene expression data of brain lower grade gliomas and glioblastoma from The Cancer Genome Atlas (TCGA) data portal (https://tcga-data.nci.nih.gov/tcga/). The TCGA lower grade glioma cohort (TCGA LGG) comprised expression profiles of 465 patients measured on Agilent 244K Custom Gene Expression G4502A-07-3 microarrays. Each patient-specific expression profile contained 15,924 genes that were overlapping with the 16,973 genes contained in our basic astrocytoma data sets described in the main manuscript. The gene expression measurements were provided as log-ratios of tumor compared to a normal universal reference. The TCGA LGG cohort included 50 AS II and 104 AS III samples. The other samples were oligoastrocytomas (OAS II: 50, OAS III: 69), oligodendrogliomas (OD II 69, OD III: 92) and no clinical information was available for 41 samples. Similarly, the TCGA glioblastoma cohort (TCGA GBM: GBM IV) comprised expression profiles of 553 GBM IV patients measured on Agilent 244K Custom Gene Expression G4502A-07-1 or G4502A-07-2 microarrays. Each patient-specific expression profile comprised 14,213 genes that overlapped with the 16,973 genes in our basic astrocytoma data sets.

1.4.2 Network validation using independent glioma cohorts

We validated the predictive power of the obtained signature-specific regulatory network on independent data. Therefore, we utilized more than one thousand publicly available gene expression profiles from three independent brain tumor cohorts (41 PA I tumors from [9], 465 low grade gliomas (including AS II and AS III) and 553 GBM IV tumors from TCGA). For each signature gene, we predicted its expression level in each of these public tumor expression profiles using the previously determined signature gene-specific linear model with corresponding parameters derived from our astrocytoma training data set. We then computed the correlation between predicted and measured expression levels of each signature gene across all tumor samples for each validation cohort and observe significant shifts of each cohort-specific correlation distribution into the positive range (Fig. S7b-d, $P < 2.2 \times 10^{-16}$ for each validation cohort, Wilcoxon rank sum test). Thus, the derived regulatory network is also highly predictive for the vast majority of signature genes in independent brain tumors suggesting that the network includes relevant regulatory links.

1.5 Text S5: Comparison of motif search-based and gene expression-based TF-target links

1.5.1 Motif search-based TF-target identification

We downloaded TF-binding site models from [11] and [12] for TFs included in the molecular signature distinguishing PA I from AS II, AS III and GBM IV. These models enabled us to compare predicted target

genes of TFs in the transcriptional regulatory network to corresponding target genes of signature-specific TFs identified using motif search to determine the proportion of putative direct physical interactions in the network. Each TF-binding site model was represented by a specific position weight matrix (PWM) modeling the DNA-binding preferences of the TF. We obtained PWMs of 28 TFs from HOCOMOCO [11] and 37 TF-specific PWMs from JOLMA [12]. We further determined putative promoter sequences (DNA sequences 1,000 bp upstream of annotated transcription start sites) of all 1,089 molecular signature genes using the UCSC Genome Browser (upstream1000.fa.gz: hg19, GRCh37, downloaded April 2014). We next used FIMO (Find Individual Motif Occurrences) [13] to search for binding sites of each signature-specific TF in the promoter sequences of all signature genes. For each TF, we provided its specific PWM and all promoter sequences of signature genes as input data to FIMO. FIMO reported by default all motif occurrences in promoter sequences with p-values less than 0.0001. This enabled us to determine putative target genes of each specific TF based on its DNA-binding preferences. We compared these independently derived TF target gene interactions with the corresponding target genes of the TF in our signature-specific transcriptional regulatory network. To realize this, we quantified the significance of the size of the overlap between both sets of target genes using Fisher's exact test.

1.5.2 Signature-specific TF-target validation

We further compared predicted target genes of TFs in the regulatory network to target genes predicted using motif search to evaluate the proportion of putative direct physical interactions in the regulatory network. Therefore, we considered binding site models of signature-specific TFs included in HOCOMOCO [11] and JOLMA [12] and searched for occurrences of putative TF binding sites in promoter sequences 1,000 bp upstream of each signature gene using FIMO [13]. This allowed us to identify potential direct target genes that we compared with the corresponding target genes of the TF in the regulatory network. We then tested for each TF if the intersection of both target gene sets was larger than expected by chance. We next plotted the cumulative p-value distributions for TFs included in HOCOMOCO and JOLMA to evaluate if small p-values are overrepresented (Fig. S8). We find clear shifts of p-values toward zero comparing network- and motif-based TF target genes against uniformly distributed p-values representing the baseline for no enrichment ($P < 0.008$ for HOCOMOCO and $P < 0.08$ for JOLMA, Wilcoxon rank sum test). For both resources, about 43% of the TFs showed an enrichment of network- and motif-based target genes with p-values below 0.1, but also about 22% of the TFs from HOCOMOCO and about 32% of the TFs from JOLMA had only very low or no overlaps between both sets of predicted target genes. Overall, this still indicates that the majority of the evaluated network-based TF target interactions may indeed represent putative direct physical interactions, while other interactions may represent indirect regulatory impacts or co-expression of a TF and a signature gene.

2 Supplementary Figures

2.1 Figure S1: Age distribution of patients diagnosed with astrocytomas of different WHO grades

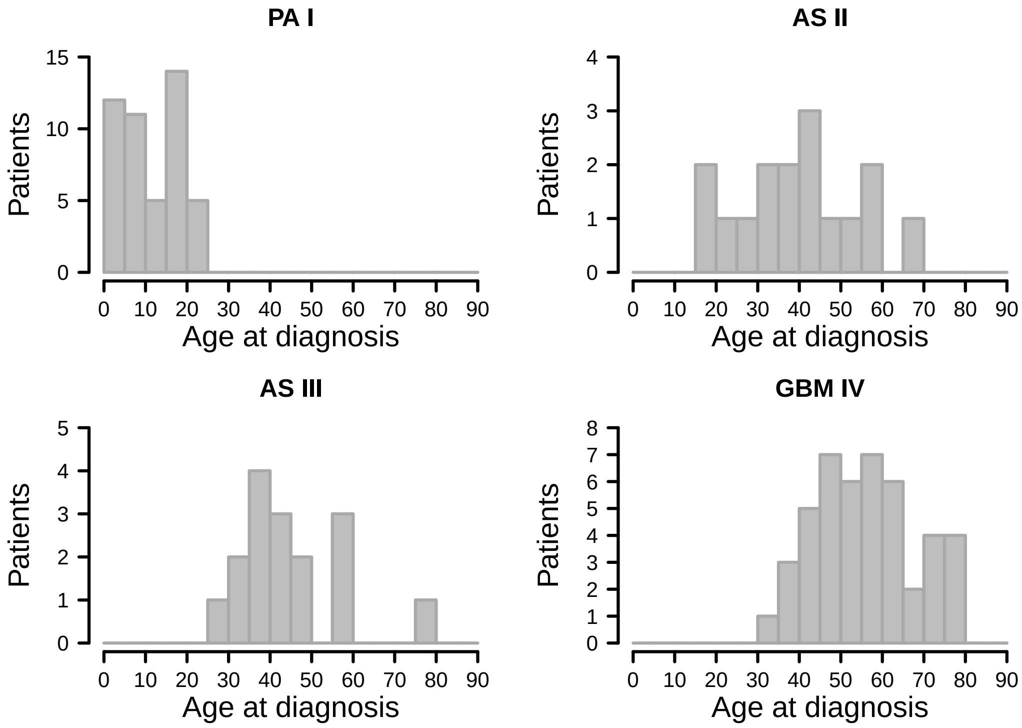

Figure S1: Age distributions of patients diagnosis with PA I, AS II, AS III and GBM IV in our data set. PA I was predominately diagnosed in children and young adults. The average diagnosis age tends to increase with the WHO grade from PA I to GBM IV.

2.2 Figure S2: Functional categorization of differentially expressed genes

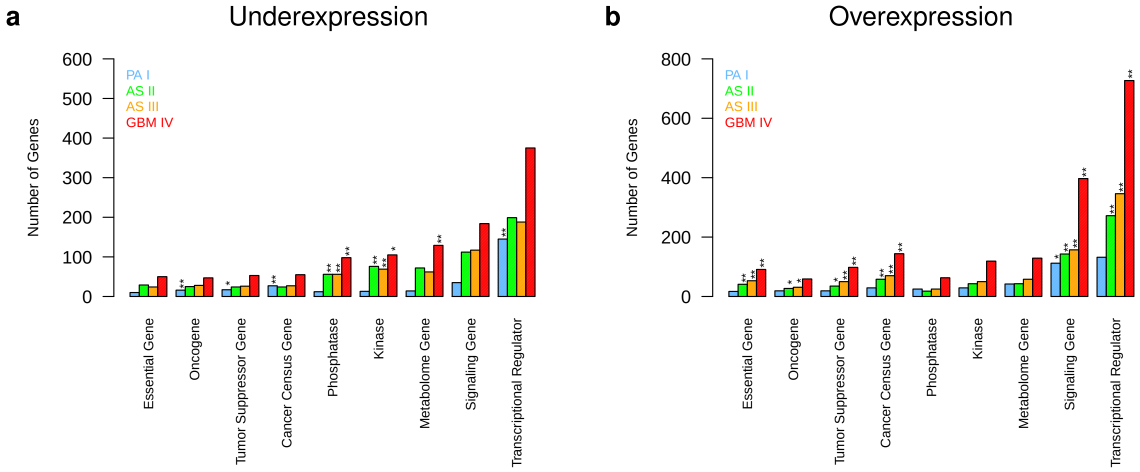

Figure S2: Functional categorization of under- and overexpressed genes for different astrocytoma grades. **a**, Number of underexpressed genes at an FDR of 0.0001 annotated in selected functional categories: essential genes, oncogenes, tumor suppressor genes, cancer census genes, phosphatases, kinases, metabolome pathway genes, signaling pathway genes, and transcriptional regulators (see Methods for details). Significant enrichment of genes in a category within a tumor type is represented by '*' ($P < 0.05$) and '**' ($P < 0.01$) (Fisher's exact test). **b**, As in **a**, but now for overexpressed genes.

2.3 Figure S3: Verhaak-subtype classification of two independent PA I cohorts

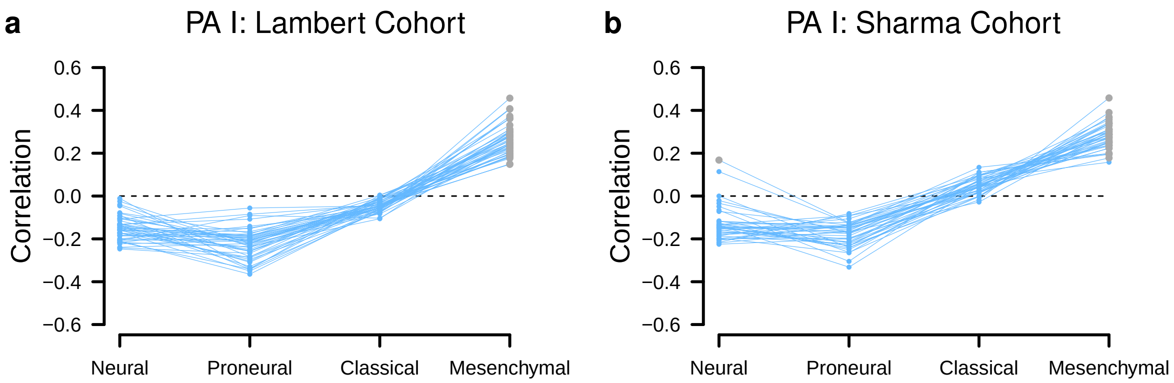

Figure S3: Verhaak-classification of individual PA I tumors of two independent cohorts. Correlations between patient-specific expression levels of Verhaak signature genes and each of the four subtype-specific Verhaak signatures were computed for each patient. Colored curves represent the obtained patient-specific correlations. A grey dot within each patient-specific curve highlights the assigned Verhaak-subtype to which the underlying patient had the strongest positive correlation. **a**, PA I cohort from Lambert *et al.* (2013) [14] intensively studied in the main manuscript. **b**, PA I cohort from Sharma *et al.* (2007) [9] under consideration of normal cerebellum references from [14].

2.4 Figure S4: Ang2 expression in endothelial cells in PA I and GBM IV tumors

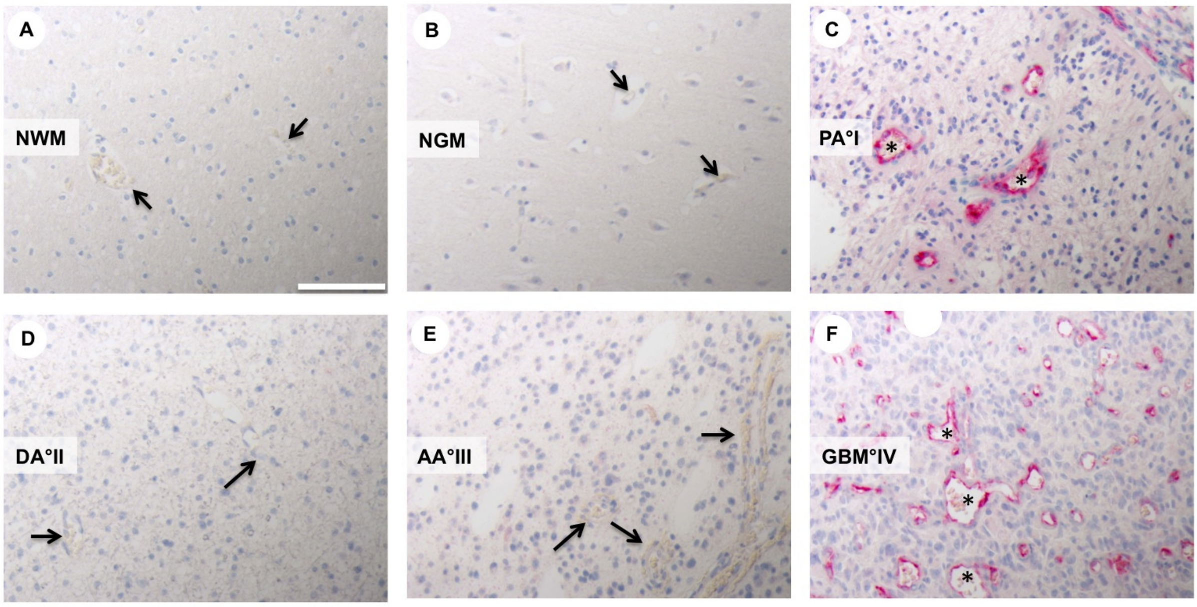

Figure S4: Ang2 immunhistochemistry in (A) normal white matter (NWM), (B) normal gray matter (NGM), (C) pilocytic astrocytoma (PA I), (D) diffuse astrocytoma (AS II), (E) anaplastic astrocytoma (AS III) and (F) glioblastoma (GBM IV). While almost no Ang2 expression was seen in (A) NWM, (B) NGM, (D) AS II and (E) AS III neither on tumor nor on endothelial (arrows) cells, prominent Ang2 expression was seen on endothelial cells of most activated blood vessels (asterisks) in both (C) PA I and (F) GBM IV. Scale bar in A = 100µm. See Text S3 for details.

2.5 Figure S5: Associations of astrocytomas with hypermethylator subtype

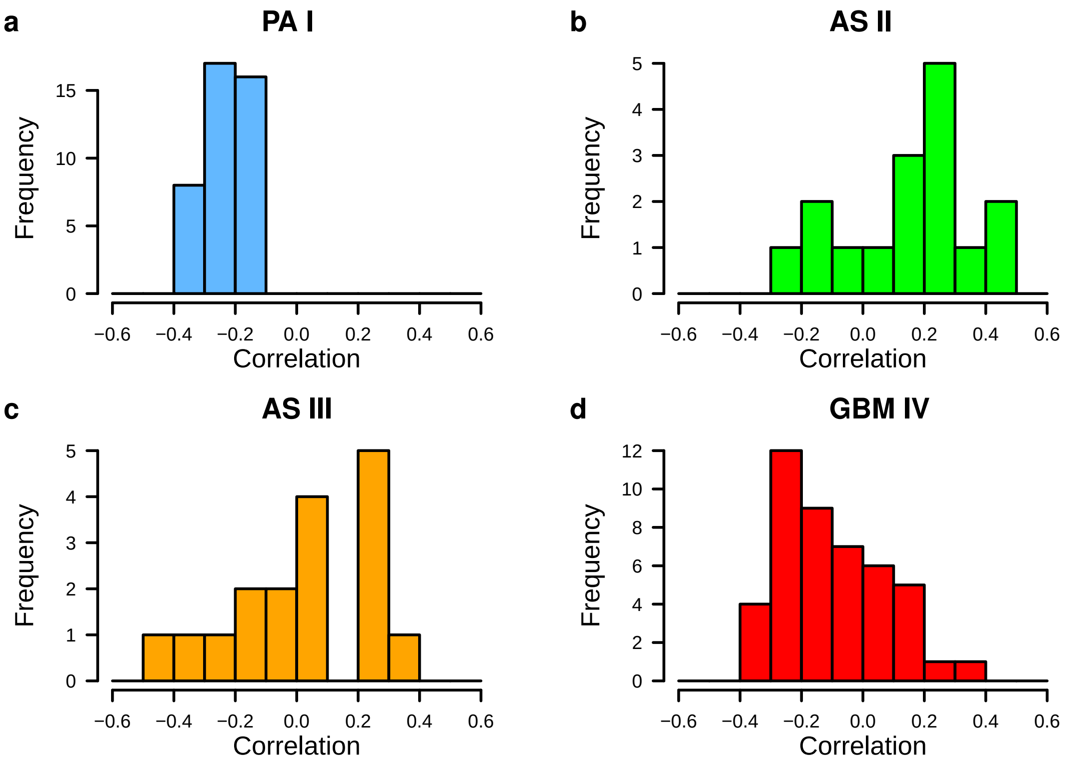

Figure S5: Correlations of individual astrocytomas with the hypermethylator phenotype. The gene expression signature characterizing the hypermethylator subtype was taken from Tab. 2 in [15]. Correlations between astrocytoma-specific expression levels of hypermethylator signature genes and the corresponding hypermethylator-specific expression levels were computed for each astrocytoma. The subfigures **a** to **d** show the correlations obtained for our PA I, AS II, AS III, and GBM IV samples. A strong negative correlation indicates that a tumor does not show the hypermethylator phenotype, whereas a positive correlations indicates that a tumor shows the hypermethylator phenotype.

2.6 Figure S6: Differential expression in individual signaling pathways

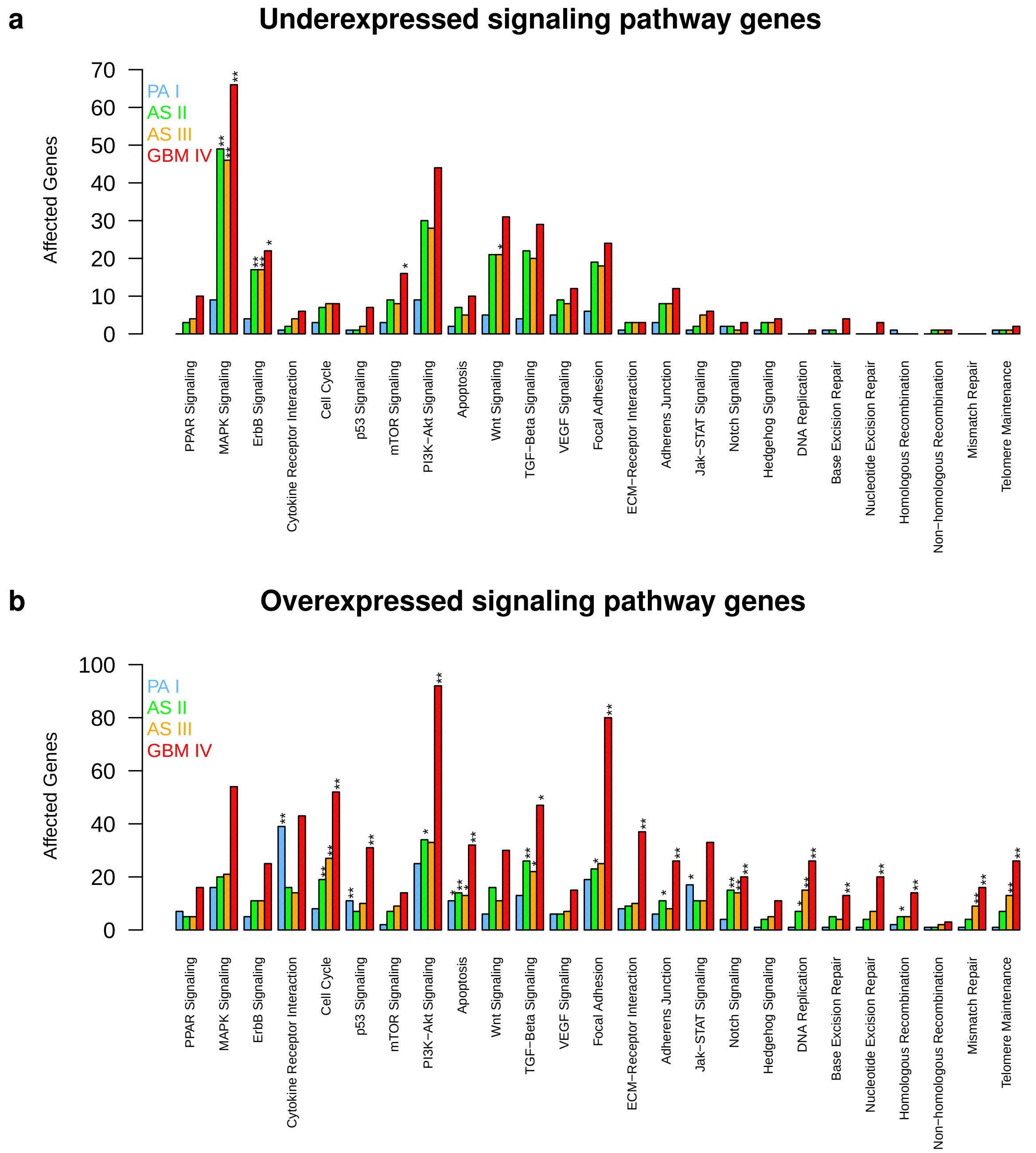

Figure S6: Numbers of under- and overexpressed genes in each known cancer-relevant signaling pathway are shown for each type of astrocytoma (PA I, AS II, AS III, GBM IV). Significant enrichment of differentially expressed genes in a pathway within a tumor type is highlighted by '*' ($P < 0.05$) and '**' ($P < 0.01$) (Fisher's exact test). **a**, Underexpressed signaling pathway genes. **b**, Overexpressed signaling pathway genes.

2.7 Figure S7: Validation of the regulatory network inferred from the molecular signature distinguishing PA I from AS II, AS III and GBM IV

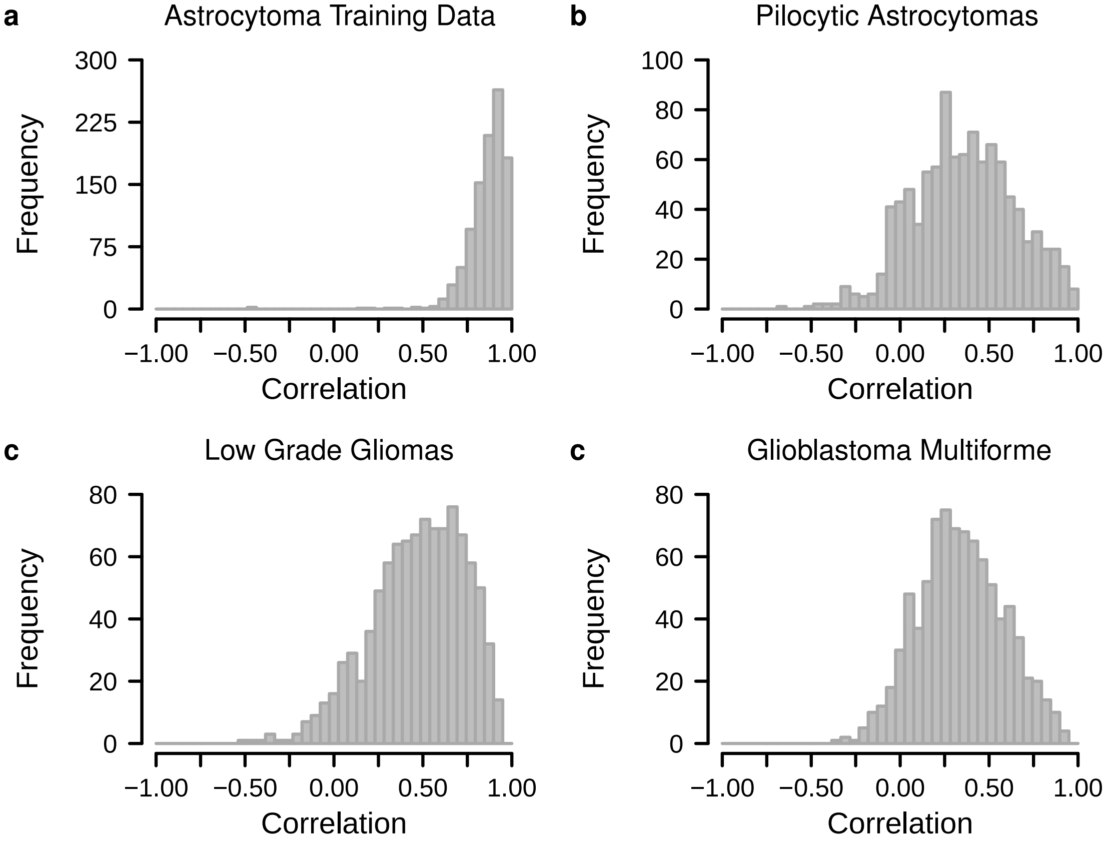

Figure S7: Correlations between signature gene-specific network-predicted and originally measured expression levels of individual genes. **a**, Performance on astrocytoma training data. **b** to **d**, Same as in sub-panel **a**, but now for independent validation data sets. **b**, Performance on 41 pilocytic astrocytomas from [9]. **c**, Performance on 465 TCGA low grade gliomas. **d**, Performance on 553 TCGA glioblastoma samples. See Text S4 for details.

2.8 Figure S8: Overlap of network-based TF-target gene interactions and motif-based TF-binding sites

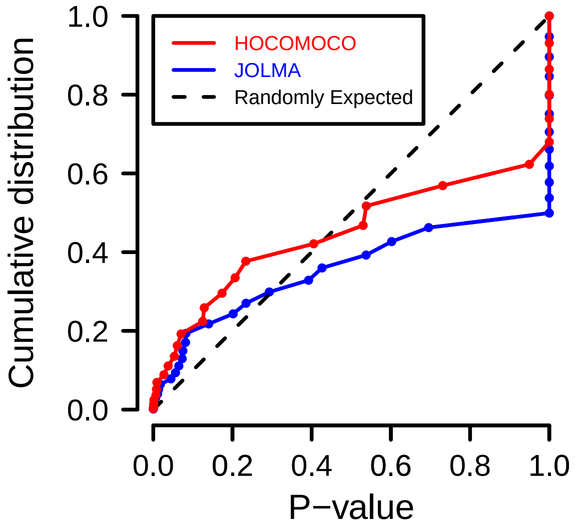

Figure S8: Comparison of expression-based predicted and via motif search identified TF-target interactions associated with the molecular signature distinguishing PA I from AS II, AS III and GBM IV. Fisher's exact test was used to quantify if the overlap of both independently derived TF-specific target gene sets was greater than randomly expected. Cumulative distribution functions of corresponding p-values are shown for signature-specific TFs from HOCOMOCO [11] (red) and JOLMA [12] (blue). The black dashed line represents the baseline for a uniform distribution of p-values. See Text S5 for details.

2.9 Figure S9: TF-TF-interaction network distinguishing PA I from AS II, AS III and GBM IV

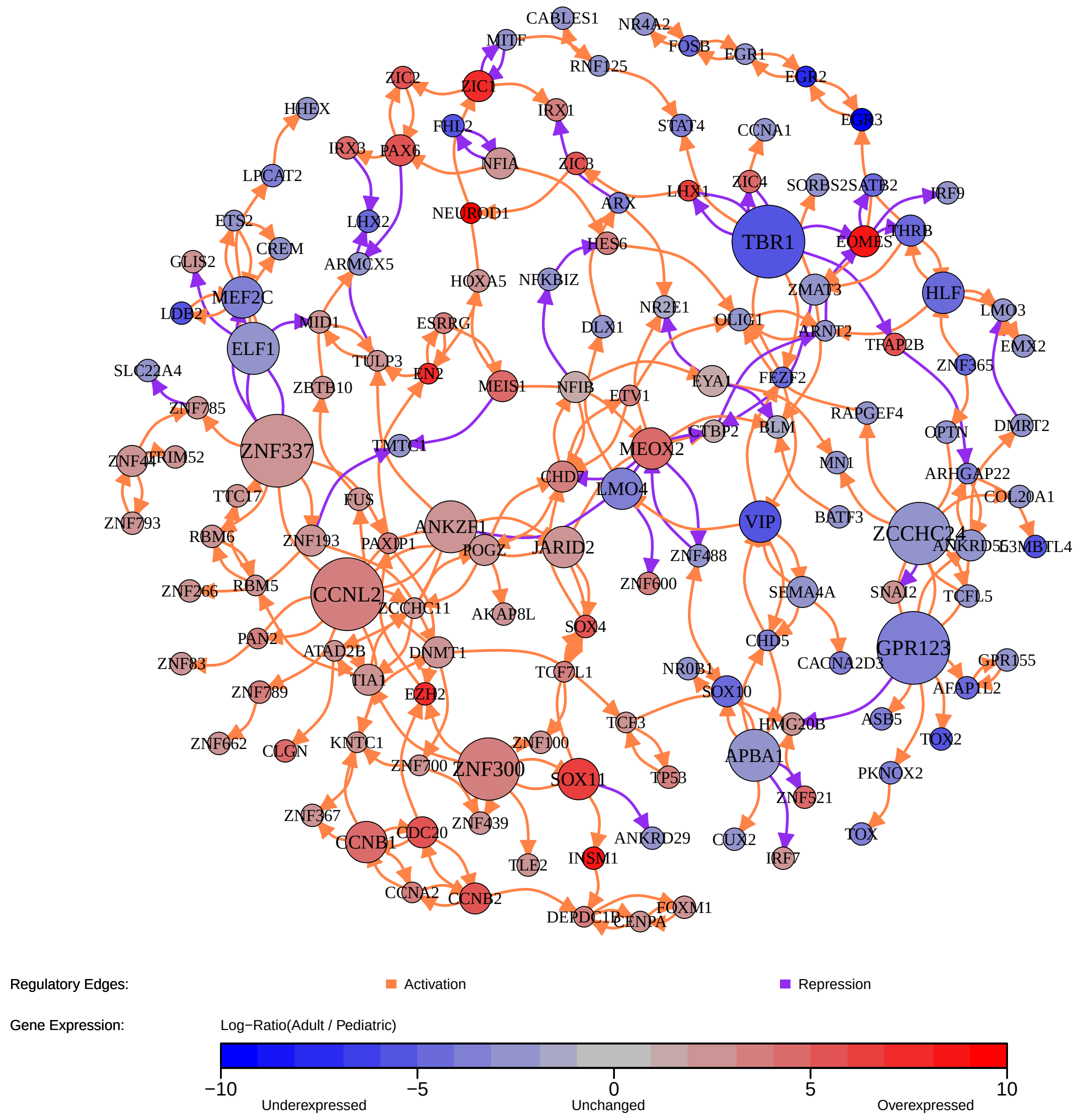

Figure S9: TF-TF-interaction network distinguishing PA I from AS II, AS III and GBM IV. TFs are displayed by circles. The circle size increases with the number of outgoing regulatory edges to other TFs highlighting major regulators by large circles. The color coding of the circle represents the average expression level of the TF across AS II, AS III and GBM IV diagnosed in adults relative to PA I diagnosed in children and young adults: underexpressed (blue) and overexpressed (red) in adult astrocytomas. Inferred regulatory dependencies between individual TFs are displayed by directed edges: activator (orange) and repressor (purple).

References

[1] S. Madhavan, J.-C. Zenklusen, Y. Kotliarov, H. Sahmi, H. A. Fine, and K. Buetow. Rembrandt: Helping personalized medicine become a reality through integrative translational research. *Mol. Cancer Res.*, 7:157–167, 2009.

[2] M. Reich, T. Liefeld, J. Gould, J. Lerner, P. Tamayo, and J. P. Mesirov. GenePattern 2.0. *Nature Genet*, 38(5):500–501, 2006.

[3] E. S. Venkatraman and A. B. Olshen. A faster circular binary segmentation algorithm for the analysis of array CGH data. *Bioinformatics*, 23(6):657–663, 2007.

[4] R. Tibshirani. Regression shrinkage and selection via the lasso. *J R Stat Soc Series B*, 58:267–288, 1996.

[5] J. Friedman, T. Hastie, and R. Tibshirani. Regularization paths for generalized linear models via coordinate descent. *J Stat Softw*, 33:1–22, 2010.

[6] R. Lockhart, J. Taylor, R. J. Tibshirani, and R. Tibshirani. A significance test for the lasso. *Ann. Stat.*, 42:413–468, 2014.

[7] T. Hastie and B. Efron. LARS: Least Angle Regression, Lasso and Forward Stagewise,. R package version 1.2, 2013.

[8] R. Lockhart, J. Taylor, R. Tibshirani, and R. Tibshirani. covTest: Computes covariance test for adaptive linear modelling,. R package version 1.01, 2013.

[9] M. K. Sharma, D. B. Mansur, G. Reifenberger, A. Perry, J. R. Leonard, K. D. Aldape, et al. Distinct genetic signatures among pilocytic astrocytomas relate to their brain region origin. *Cancer Res.*, 67:890–900, 2007.

[10] Z. Wu, R. A. Irizarry, R. Gentleman, F. Martinez-Murillo, and F. Spencer. A model-based background adjustment for oligonucleotide expression arrays. *J. Am. Statist. Assoc.*, 99:909–917, 2004.

[11] I. V. Kulakovskiy, Y. A. Medvedeva, U. Schaefer, A. S. Kasianov, I. E. Vorontsov, V. B. Bajic, and V. J. Makeev. HOCOMOCO: a comprehensive collection of human transcription factor binding sites models. *Nucl. Acids Res.*, 41:D195–D202, 2013.

[12] A. Jolma, J. Yan, T. Whitington, J. Toivonen, K. R. Nitta, P. Rastas, et al. DNA-binding specificities of human transcription factors. *Cell*, 152(1-2):327–339, 2013.

[13] C. E. Grant, T. L. Bailey, and W. S. Noble. FIMO: Scanning for occurrences of a given motif. *Bioinformatics*, 27:1017–1018, 2011.

[14] S. R. Lambert, H. Witt, V. Hovestadt, M. Zucknick, M. Kool, D. M. Pearson, et al. Differential expression and methylation of brain developmental genes define location-specific subsets of pilocytic astrocytoma. *Acta Neuropathol.*, 126:291–301, 2013.

[15] H. Noushmehr et al. Identification of a CpG island methylator phenotype that defines a distinct subgroup of glioma. *Cancer Cell*, 17:510–522, 2010.
